# Single Dose of Amphetamine Induces Delayed Subregional Attenuation of Cholinergic Interneuron Activity in the Striatum

**DOI:** 10.1523/ENEURO.0196-21.2021

**Published:** 2021-09-20

**Authors:** Samira Ztaou, Soo Jung Oh, Sophia Tepler, Sixtine Fleury, Miriam Matamales, Jesus Bertran-Gonzalez, Nao Chuhma, Stephen Rayport

**Affiliations:** 1 Department of Molecular Therapeutics, New York State Psychiatric Institute, New York, NY 10032; 2 Department of Psychiatry, Columbia University, New York, NY 10032; 3 Decision Neuroscience Laboratory, School of Psychology, University of New South Wales, Sydney, NSW 2052, Australia

**Keywords:** acetylcholine, dopamine, fluorescence imaging, phosphorylated ribosomal protein S6, psychostimulant

## Abstract

Psychostimulants such as amphetamine (AMPH) target dopamine (DA) neuron synapses to engender drug-induced plasticity. While DA neurons modulate the activity of striatal (Str) cholinergic interneurons (ChIs) with regional heterogeneity, how AMPH affects ChI activity has not been elucidated. Here, we applied quantitative fluorescence imaging approaches to map the dose-dependent effects of a single dose of AMPH on ChI activity at 2.5 and 24 h after injection across the mouse Str using the activity-dependent marker phosphorylated ribosomal protein S6 (p-rpS6^240/244^). AMPH did not affect the distribution or morphology of ChIs in any Str subregion. While AMPH at either dose had no effect on ChI activity after 2.5 h, ChI activity was dose dependently reduced after 24 h specifically in the ventral Str/nucleus accumbens (NAc), a critical site of psychostimulant action. AMPH at either dose did not affect the spontaneous firing of ChIs. Altogether this work demonstrates that a single dose of AMPH has delayed regionally heterogeneous effects on ChI activity, which most likely involves extra-Str synaptic input.

## Significance Statement

Using the activity dependent marker phosphorylated ribosomal protein S6 (p-rpS6^240/244^), we mapped amphetamine (AMPH) effects on the activity of cholinergic interneurons (ChIs) across the striatum (Str). AMPH reduced ChI activity in dose-dependent manner in the ventral Str/nucleus accumbens (NAc), a critical site of psychostimulant action.

## Introduction

Psychostimulants such as amphetamine (AMPH) target dopamine (DA) neuron terminals (Sulzer, 2011) and engender dose-dependent behavioral effects. DA release in the ventral striatum/nucleus accumbens (Str/NAc) is associated with hyperlocomotion, whereas DA release in the dorsal Str is associated with stereotypies (Robinson and Becker, 1986; Kalivas and Stewart, 1991; Gaytan et al., 1998; Yates et al., 2007). DA neurons modulate the activity of cholinergic interneurons (ChIs), which comprise <2% of striatal (Str) neurons, and yet strongly control the Str circuitry (Goldberg and Wilson, 2010; Gonzales and Smith, 2015; Abudukeyoumu et al., 2019). Modulation of ChI activity is critical for the processing and reinforcement of reward-related behaviors (Atallah et al., 2014; Gonzales and Smith, 2015). ChIs in the ventral Str are crucial for psychostimulant-dependent behaviors (Sofuoglu and Mooney, 2009; Witten et al., 2010; Lee et al., 2020; Lewis and Borrelli, 2020). However, whether AMPH has subregional effects on ChI activity has not been elucidated.

Previous studies have shown that the phosphorylated form of the ribosomal protein S6 at serine 240 and 244 residues (p-rpS6^240/244^) reports activity of ChIs under different pharmacological and/or behavioral conditions (Bertran-Gonzalez et al., 2012; Kharkwal et al., 2016; Matamales et al., 2016a,b). The phosphorylation of rpS6 can be induced by multiple signaling cascades; mTORC1 pathway and/or mTORC1-independent pathways such as the PKC, the MAPK or the cAMP/PKA pathways (Valjent et al., 2011; Bertran-Gonzalez et al., 2012; Gangarossa and Valjent, 2012). The phosphorylation of rpS6 appears to occur sequentially at five serine residues: in the order 236, 235, 240, 244, and 247 (Knight et al., 2012; Biever et al., 2015a). Bertran-Gonzalez and colleagues showed a clear p-rpS6^240/244^ signal preferentially expressed in ChIs, in contrast to a much weaker signal of p-rpS6^235/236^ (Bertran-Gonzalez et al., 2012). Pharmacological modification of ChI firing leads to changes of p-rpS6^240/244^ intensity in ChIs (Bertran-Gonzalez et al., 2012; Matamales et al., 2016b). To address regionality in AMPH modulation of ChI activity, we mapped p-rpS6^240/244^ intensity in ChIs throughout the entire rostrocaudal axis of the Str after a single low-dose or high-dose of AMPH at two time points: 2.5 h postinjection (2.5h_pi_) and 24 h postinjection (24h_pi_). This revealed that AMPH induces a delayed regionally heterogeneous dose-dependent attenuation of ChI activity in the ventral Str/NAc.

## Materials and Methods

### Ethics

This research was performed in accordance with the *Guide for the Care and Use of Laboratory Animals* of the National Institutes of Health, under a protocol approved by the Institutional Animal Care and Use Committee of New York State Psychiatric Institute (#NYSPI-1494).

### Experimental animals

Mice were 129 Sv/C57BL6J mixed background, backcrossed to C57BL6J at least five times and kept inbred. Mice were group housed and maintained on a 12/12 h light/dark cycle with lights on at 7 A.M. in a temperature-controlled room with food and water provided *ad libitum*. The DAT-IRES-Cre/+;ROSA26-flox-STOP-CAG-ChR2-YFP double mutant strain (The Jackson Laboratory, RRID:IMSR_JAX:006660, RRID:IMSR_JAX:024109) were used, with the same genotype as previous studies (Chuhma et al., 2014, 2018; Mingote et al., 2015, 2017). The presence of Cre is not essential for the present study; the IRES-cre transgene insertion in the DA transporter (DAT) locus modestly reduces DAT expression and AMPH responsiveness (Bäckman et al., 2006; Chohan et al., 2020).

For the immunocytochemistry experiments, 30 mice were used at postnatal day (P)56–P82, divided in two cohorts of 15 for 2.5h_pi_ and 15 for 24h_pi_. Cohorts were balanced for sex: 16 male (2.5h_pi_ cohort: saline, *n* = 3; low-dose AMPH, *n* = 3; high-dose AMPH, *n* = 3 and 24h_pi_ cohort: saline, *n* = 2; low-dose AMPH, *n* = 2; high-dose AMPH, *n* = 3) and 14 female (2.5h_pi_ cohort: saline, *n* = 2; low-dose AMPH, *n* = 2; high-dose AMPH, *n* = 2 and 24h_pi_ cohort: saline, *n* = 3; low-dose AMPH, *n* = 3; high-dose AMPH, *n* = 2) mice. For the electrophysiological experiments, 40 male (saline, *n* = 17; low-dose AMPH, *n* = 11; high-dose AMPH, *n* = 12) and 40 female (saline, *n* = 13; low-dose AMPH, *n* = 11; high-dose AMPH, *n* = 16) mice at P52–P72 were used. No sex differences were observed, so data from male and female mice in each group were combined.

### Drug treatment

D-AMPH hemisulfate (Sigma-Aldrich, A5880) either low-dose (2 mg/kg) or high-dose (16 mg/kg) was dissolved in 0.9% NaCl immediately before use. Injections were done intraperitoneally at a volume of 10 ml/kg body weight.

### Behavioral monitoring

Mice were habituated to handling for 2 d before the drug administration. Monitoring took place under bright ambient light conditions during the light phase. On the injection day, mice were placed in the open field, equipped with infrared motion detectors (Plexiglas activity chambers, 40.6 cm long × 40.6 cm wide × 38.1 cm high; SmartFrame Open Field System, Kinder Scientific) for 1 h for habituation. Baseline activity was monitored for 30 min preinjection, then mice were injected intraperitoneally either with saline, 2 or 16 mg/kg AMPH, and observed for a 2-h postinjection period. Locomotor activity was recorded automatically in 10-min bins. Stereotyped behaviors, orofacial stereotypy (mouth movements, lick, bite, self-gnaw, taffy pull, jaw tremor, yawn) and grooming, were scored for 1 min every 5 min as previously described (Kelley, 2001).

### Immunocytochemistry

For immunocytochemistry, mice were deeply anesthetized with ketamine (90 mg/kg)/xylazine (7 mg/kg) and then perfused intracardially with cold PBS (100 mm; pH 7.4) followed by 4% paraformaldehyde (PFA). Brains were removed and postfixed overnight in 4% PFA. Coronal sections were cut at 50 μm with a vibrating microtome (Leica VT1200S), and stored in a cryoprotectant solution (30% glycerol, 30% ethylene glycol in 0.1 M Tris HCl, pH 7.4) at −20°C. Free-floating sections were washed in PBS and incubated in glycine (100 mM) for 30 min to quench aldehydes. Non-specific binding was blocked with 10% normal donkey serum (NDS) in 0.1 PBS Triton X-100 for 2 h. Sections were incubated in PBS containing 0.02% Triton X-100 and 2% NDS overnight at 4°C with primary antibodies: anti-ChAT (1:500, goat polyclonal, Millipore catalog #AB144P, RRID:AB_2079751) and anti-phosphorylated ribosomal protein S6 (p-rpS6^240/244^, 1:1500, rabbit polyclonal, Cell Signaling Technology catalog #2215, RRID:AB_331682). Sections were then washed with PBS, and secondary antibodies applied for 45 min in PBS containing 0.02% Triton X-100 at room temperature: anti-goat Alexa Fluor 594 (1:200, Thermo Fisher Scientific catalog #A-11 058, RRID:AB_2534105) and anti-rabbit Alexa Fluor 488 (1:200, Thermo Fisher Scientific catalog #A-21206, RRID:AB_2535792). Sections were mounted on gelatin subbed slides (SouthernBiotech) and coverslipped with ProLong Gold aqueous medium with DAPI (Thermo Fisher Scientific) and stored at 4°C until imaging.

### Imaging and analysis

Images were acquired using an Axio Imager M2 fluorescence microscope (Zeiss) with a high-resolution digital camera (Axiocam 506 mono, 2752 × 2208 pixels, Zeiss), a 20×/0.8 objective and Zen 2.3 Digital Imaging software (Zeiss; RRID:SCR_013672). Ten coronal sections, spanning the rostrocaudal extent of the right Str (bregma 1.54, 1.18, 0.98, 0.62, 0.26, −0.10, −0.46, −0.82, −1.22, and −1.58 mm), were imaged. An image stack consisting of 5 planes at 5 μm intervals was obtained. Exposure time for each excitation was held constant throughout acquisition.

Raw 16-bit images were analyzed using Fiji/ImageJ (version 2.0.0., NIH, RRID:SCR_002285). Z-projected images were obtained by taking pixels with the maximum intensity in a stack. The outer boundary of the Str and its anatomic subregions, NAc core and shell, dorsomedial (DM) Str and dorsolateral (DL) Str, were manually delineated in accordance with the mouse brain atlas (Paxinos and Franklin, 2008), and their areas (mm^2^) in each coronal section were obtained.

Particle analysis detected all ChAT-positive neurons in the Str and the total number of ChIs, perimeter (μm), area (μm^2^), and circularity (a circularity value of 1 indicates a perfect circle while values approaching 0 indicate more elongated shapes) of each ChI were measured. Density of ChIs (neurons/mm^2^) in each Str subregion was calculated as ChI number in a subregion divided by area of the subregion. For each coronal section, the ChAT image was superimposed on the p-rpS6^240/244^ image, and the ChAT-positive neurons were used as a mask for p-rpS6^240/244^ intensity analysis. Fluorescence intensity of the corpus callosum was used for background subtraction.

Location of each ChI was defined by coordinates of the centroid. The p-rpS6^240/244^ intensity of each ChI was normalized to the maximum and the minimum intensities for each cohort, 2.5h_pi_ or 24h_pi_, and color-scaled. All color-scaled ChIs were 3D plotted with outlines of the Str using a customized script in MATLAB (MathWorks; RRID:SCR_001622) as previously described (Matamales et al., 2016a).

Distributions of p-rpS6^240/244^ fluorescence intensity were standardized to the corresponding saline group for each time point and subregion by calculating z-scores: z = (x – μ)/σ, where x is the p-rpS6^240/244^ signal in individual ChIs, μ and σ are the mean and the SD, respectively, of p-rpS6^240/244^ signal in the corresponding saline group.

### Slice electrophysiology and analysis

For electrophysiology recording, mice were anesthetized with ketamine (90 mg/kg)/xylazine (7 mg/kg). After confirmation of full anesthesia, mice were decapitated and brains quickly removed in ice-cold high-glucose artificial CSF (ACSF; 75 mM NaCl, 2.5 mM KCl, 25 mM NaHCO_3_, 1.25 mM NaH_2_PO_4_, 0.7 mM CaCl_2_, 2 mM MgCl_2_, and 100 mM glucose; pH 7.4) saturated with carbogen (95% O_2_ + 5% CO_2_). Coronal sections of the Str (bregma from 1.70 to 0.26 mM) were cut, 300 μm thick, with a vibrating microtome (VT1200S, Leica), incubated in high-glucose ACSF at room temperature for at least 1 h for recovery, then transferred to the recording chamber (submerged, 500 μl volume) on the stage of an upright microscope (BX61WI, Olympus), continuously perfused with standard ACSF (125 mM NaCl, 2.5 mM KCl, 25 mM NaHCO_3_, 1.25 mM NaH_2_PO_4_, 2 mM CaCl_2_, 1 mM MgCl_2_, and 25 mM glucose; pH 7.4) saturated with carbogen. Recorded neurons were visualized using enhanced visible light differential interference contrast (DIC) optics with a scientific c-MOS camera (ORCA-Flash4.0LT, Hamamatsu Photonics).

ChIs were identified visually by large soma size, confirmed by spontaneous firing, shallow resting membrane potentials (around 60 mV) and voltage sag by −400 pA current injection (700 ms in duration; Chuhma et al., 2014, 2018). Recording patch pipettes were fabricated from standard-wall borosilicate glass capillary with filament (World Precision Instruments). Pipette resistance was 4–9 MΩ and series resistance was 7–32 MΩ. Composition of intracellular solution was 135 mM K^+^-methane sulfonate (MeSO_4_), 5 mM KCl, 2 mM MgCl_2_, 0.1 mM CaCl_2_, 10 mM HEPES, 1 mM EGTA, 2 mM ATP, and 0.1 mM GTP; pH 7.25. Recording was done with an Axopatch 200B amplifier (Molecular Devices) in fast current clamp mode. All recordings were done at 32–34°C (TC 344B Temperature Controller, Warner Instruments). No more than four cells were recorded per animal.

Data were filtered at 5 kHz using a four-pole Bessel filter, digitized at 5 kHz (Digidata 1550A, Molecular Devices) and recorded using pClamp 10 (Molecular Devices; RRID:SCR_011323). Electrophysiological data were analyzed with Axograph X (Axograph Science; RRID:SCR_014284). Firing frequencies were calculated as average frequency in a 2 s window obtained from 10 consecutive traces.

### Statistical analysis

Sample sizes were determined using G*Power 3.1 with effect sizes based on similar experiments (G*Power, RRID: SCR_013726), setting α = 0.05 and power = 0.8 (Cunningham and McCrum-Gardner, 2007; Faul et al., 2007). For the immunocytochemistry experiments, we used Cohen’s *d* = 0.97 as an effect size, resulting in 5 mice per group. For the electrophysiological experiments, we used Cohen’s *d* = 0.32 as an effect size, resulting in 12 mice per group.

Statistical analyses were performed using Prism 8 (GraphPad Prism, RRID:SCR_002798) or SPSS 26 (SPSS; RRID:SCR_002865). *p* < 0.05 was considered as significant for all analyses. Data are presented as mean ± SEM. Parametric tests were used here because datasets followed a normal distribution (D’Agostino–Pearson normality test, *p* > 0.05). ANOVA was used for comparison among conditions. Where significance was detected, multiple pairwise comparisons with Bonferroni correction were performed as *post hoc* tests.

## Results

### Dose-dependent effects of AMPH on locomotor activity and stereotypy

Behavioral observations were used to confirm the dose-dependent effects of AMPH. Mice received a single low-dose (2 mg/kg) or high-dose (16 mg/kg) of AMPH, and their brains were extracted for analysis either after 2.5h_pi_, when acute behavioral effects had subsided, or at 24h_pi_ to assess enduring effects on ChI activity in the Str. One low-dose AMPH-injected mouse, in the 2.5h_pi_ cohort, was excluded from the study as its locomotor activity decreased after injection.

**Figure 1.**
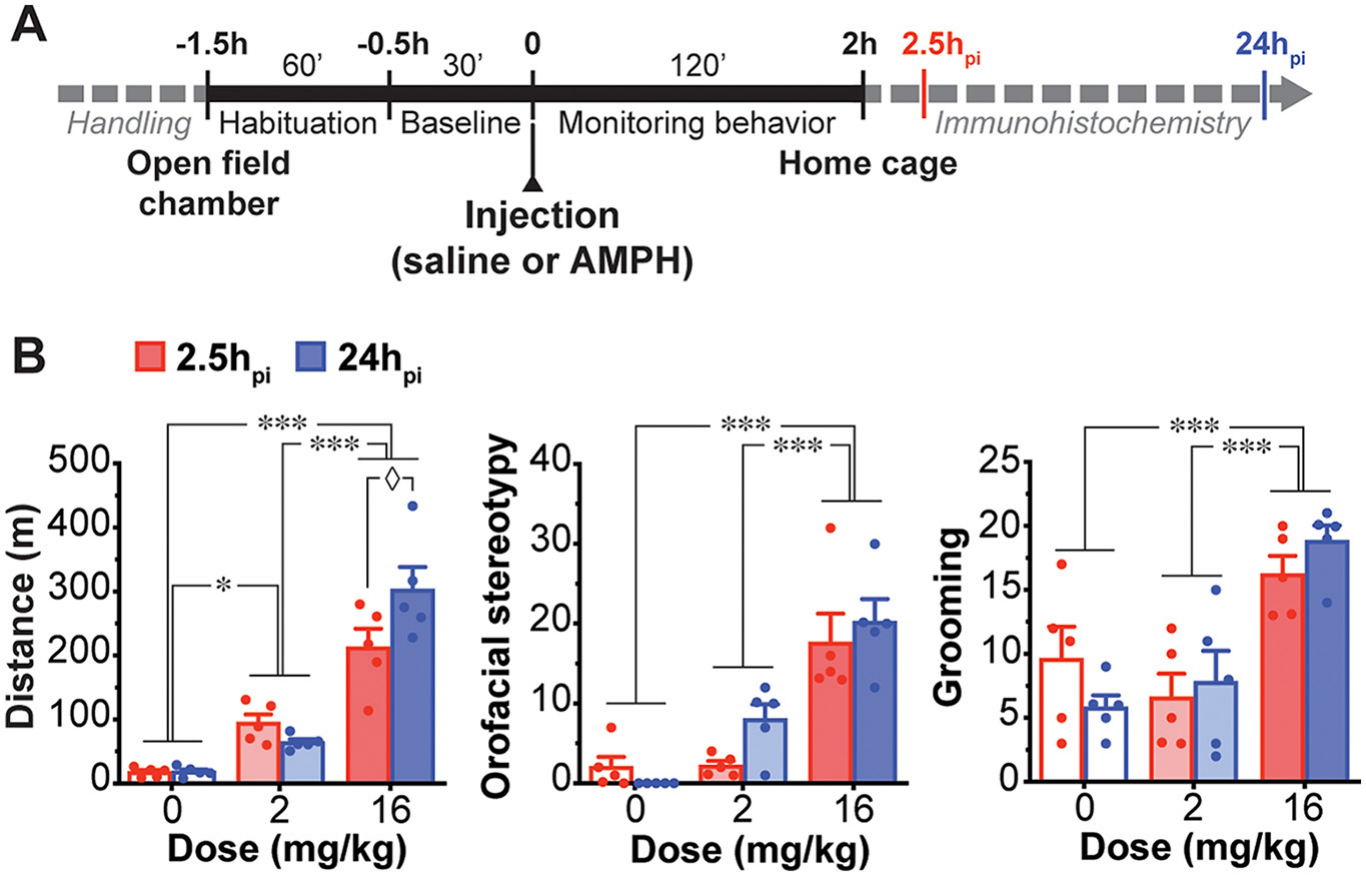
Dose-dependent behavioral effects of AMPH. ***A***, Timeline of AMPH experiments. ***B***, Total distance traveled (left), orofacial stereotypy (middle), and grooming (right) scores are shown after saline (0 mg/kg, *n* = 5 animals), low-dose (2 mg/kg, *n* = 5 animals), or high-dose (16 mg/kg, *n* = 5 animals) AMPH, for 2.5h_pi_ cohort (red) and 24h_pi_ cohort (blue). Dots in bar graphs show measurements per animal. *p < 0.05, **p < 0.01, ***p < 0.001 for comparison among doses; ◊*p* < 0.05 for comparison between 2.5h_pi_ and 24h_pi_.

To confirm differential behavioral effects of the two AMPH doses and similar behavioral effects in the two cohorts (2.5h_pi_ and 24h_pi_), mice from each cohort received saline, low-dose or high-dose AMPH, and locomotion and stereotypy were monitored for 2 h in the open field (Fig. 1*A*). Total travel distance dose dependently increased in both the 2.5h_pi_ cohort (saline 17.6 ± 3.4 m, low-dose 94.5 ± 13.8 m, high-dose 212.5 ± 29.3 m) and 24h_pi_ cohort (saline 18.6 ± 3.4 m, low-dose 64.0 ± 5.0 m, high-dose 302.7 ± 35.8 m), while no significant difference was observed between the two cohorts (two-way ANOVA; treatment effect, *F*_(2,24)_ = 78.15, *p *<* *0.001; cohort effect, *F*_(1,24)_ = 1.55, *p *=* *0.23; Fig. 1*B*, left). Although there was a significant treatment × cohort interaction (*F*_(2,24)_ = 4.95, *p *=* *0.02), the two cohorts showed similar dose-dependent hyperlocomotion, a significant increase after low-dose and a further increase after high-dose.

Low-dose and high-dose AMPH increased orofacial stereotypy in both the 2.5h_pi_ cohort (saline 2.0 ± 1.3, low-dose 2.2 ± 0.6, high-dose 17.6 ± 3.6) and 24h_pi_ cohort (saline 0 ± 0, low-dose 8.0 ± 1.9, high-dose 20.2 ± 2.9), while no significant difference was observed between the two cohorts (two-way ANOVA; treatment effect, *F*_(2,24)_ = 38.74, *p *<* *0.001; cohort effect, *F*_(1,24)_ = 1.50, *p *=* *0.23; treatment × cohort interaction, *F*_(2,24)_ = 1.69, *p *=* *0.21; Fig. 1*B*, middle).

Low-dose AMPH did not affect grooming score in the two cohorts, while high-dose increased it in both 2.5h_pi_ cohort (saline 9.6 ± 2.5, low-dose 6.6 ± 1.9, high-dose 16.2 ± 1.5) and 24h_pi_ cohort (saline 5.8 ± 0.9, low-dose 7.8 ± 2.4, high-dose 18.8 ± 1.2; two-way ANOVA; treatment effect, *F*_(2,24)_ = 19.86, *p *<* *0.001). Neither a significant difference, nor a treatment × cohort interaction, was observed between the two cohorts (cohort effect, *F*_(1,24)_ = 0, *p *>* *0.99; treatment × cohort interaction, *F*_(2,24)_ = 1.67, *p *=* *0.21; Fig. 1*B*, right). These observations confirmed that AMPH elicited a comparable dose-dependent behavioral activation in the two cohorts, used for the 2.5h_pi_ and 24h_pi_ ChI studies.

### Distribution and morphology of ChIs is not affected by AMPH

**Figure 2.**
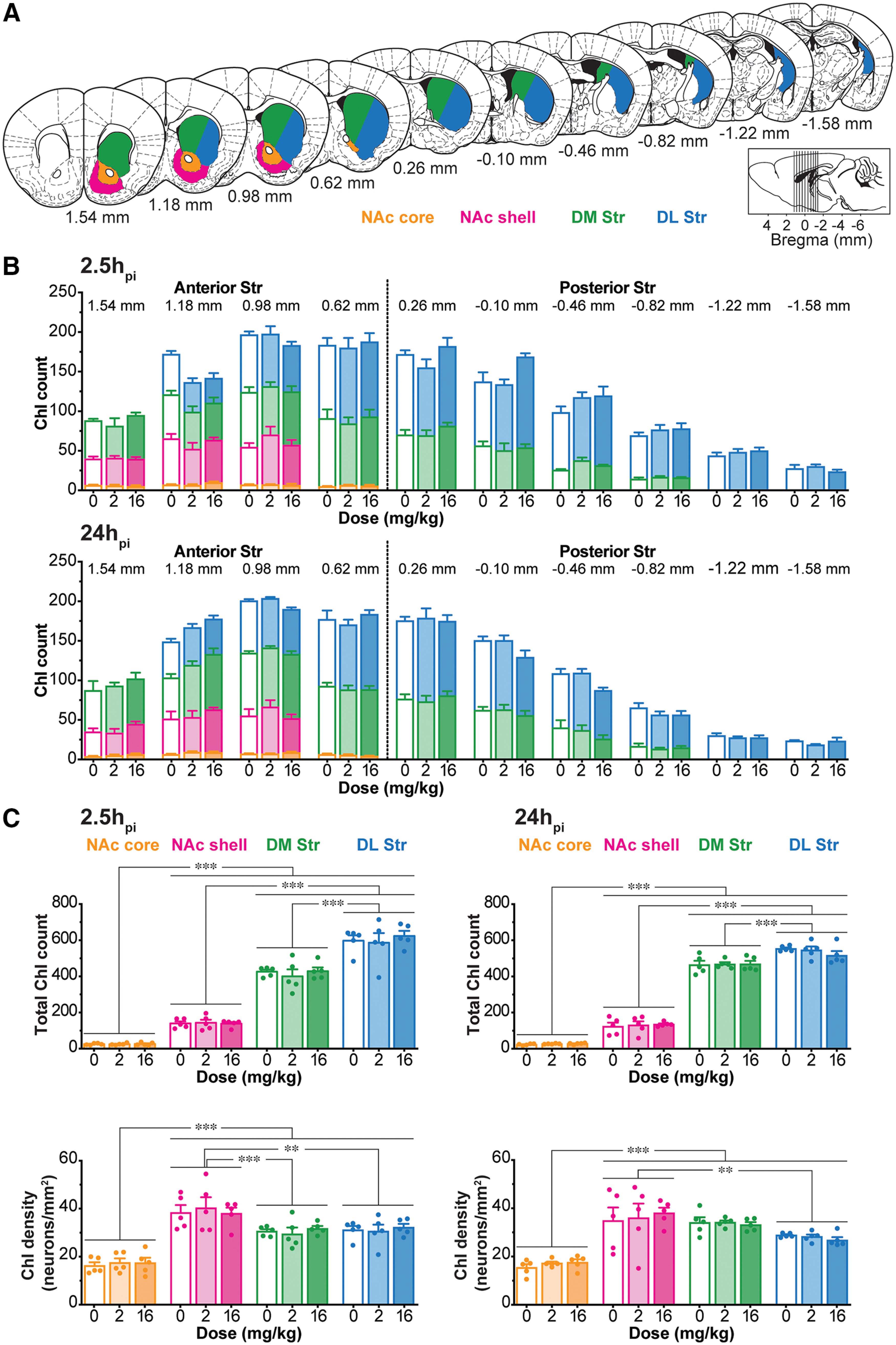
Distribution of ChIs in the Str is not affected by AMPH. ***A***, Schematic representations of 10 coronal sections of the Str (from bregma +1.54 to −1.58 mm). Delineations of Str subregions are shown in the right Str (Paxinos and Franklin, 2008): NAc core (orange), NAc shell (magenta), DM Str (green), DL Str (blue). Locations of slices are shown in inset, and locations from bregma are indicated under the slices. ***B***, Stacked bar graphs showing counts of ChIs across Str subregions in 10 coronal hemisections along the rostrocaudal axis after saline (0 mg/kg), low-dose (2 mg/kg), or high-dose (16 mg/kg) AMPH, at 2.5h_pi_ (top) and 24h_pi_ (bottom). ***C***, Total ChI count (top) and ChI density (neurons/mm^2^; bottom) in each Str subregion are shown, at 2.5h_pi_ (left) and 24h_pi_ (right). Group *n*s are given in Figure 1. Dots in bar graphs show the average per animal; **p < 0.01 and ***p < 0.001.

To address potential neurotoxic effects of AMPH on ChIs, particularly high-dose (viz., Zhu et al., 2006), we examined ChI distribution and soma morphology. ChIs were identified by ChAT immunostaining and examined in 10 coronal sections spanning the rostrocaudal extent of the right Str in four subregions: NAc core and shell, DM and DL Str (Fig. 2*A*). The previously recognized rostro-caudal distribution of ChIs (Matamales et al., 2016a) peaked at 0.98 mm from bregma and gradually declined caudally. The distribution was not affected by either AMPH dose or time after injection (three-way ANOVA; rostrocaudal effect, *F*_(9,240)_ =204.13, *p *<* *0.001; treatment effect, *F*_(2,240)_ = 0.17, *p *=* *0.85; time effect, *F*_(1,240)_ = 0.66, *p *=* *0.42; rostrocaudal × treatment × time interaction, *F*_(18,240)_ = 1.43, *p *=* *0.12; Fig. 2*B*). Although numbers of ChIs varied significantly between Str subregions, AMPH dose or time after injection did not affect ChI count significantly in any Str subregion (three-way ANOVA; location effect, *F*_(3,96)_ = 954.82, *p *<* *0.001; treatment effect, *F*_(2,96)_ = 0.12, *p *=* *0.89; time effect, *F*_(1,96)_ = 0.42, *p *=* *0.52; location × treatment × time interaction, *F*_(6,96)_ = 0.52, *p *=* *0.80; Fig. 2*C*, top). Although ChI densities varied between Str subregions, highest in the NAc shell and lowest in the NAc core, AMPH dose or time after injection did not affect densities in any subregion (three-way ANOVA; location effect, *F*_(3,96)_ = 73.76, *p *<* *0.001; treatment effect, *F*_(2,96)_ = 0.15, *p *=* *0.86; time effect, *F*_(1,96)_ = 0.57, *p *=* *0.45; location × treatment × time interaction, *F*_(6,96)_ = 0.30, *p *=* *0.94; Fig. 2*C*, bottom).

**Figure 3.**
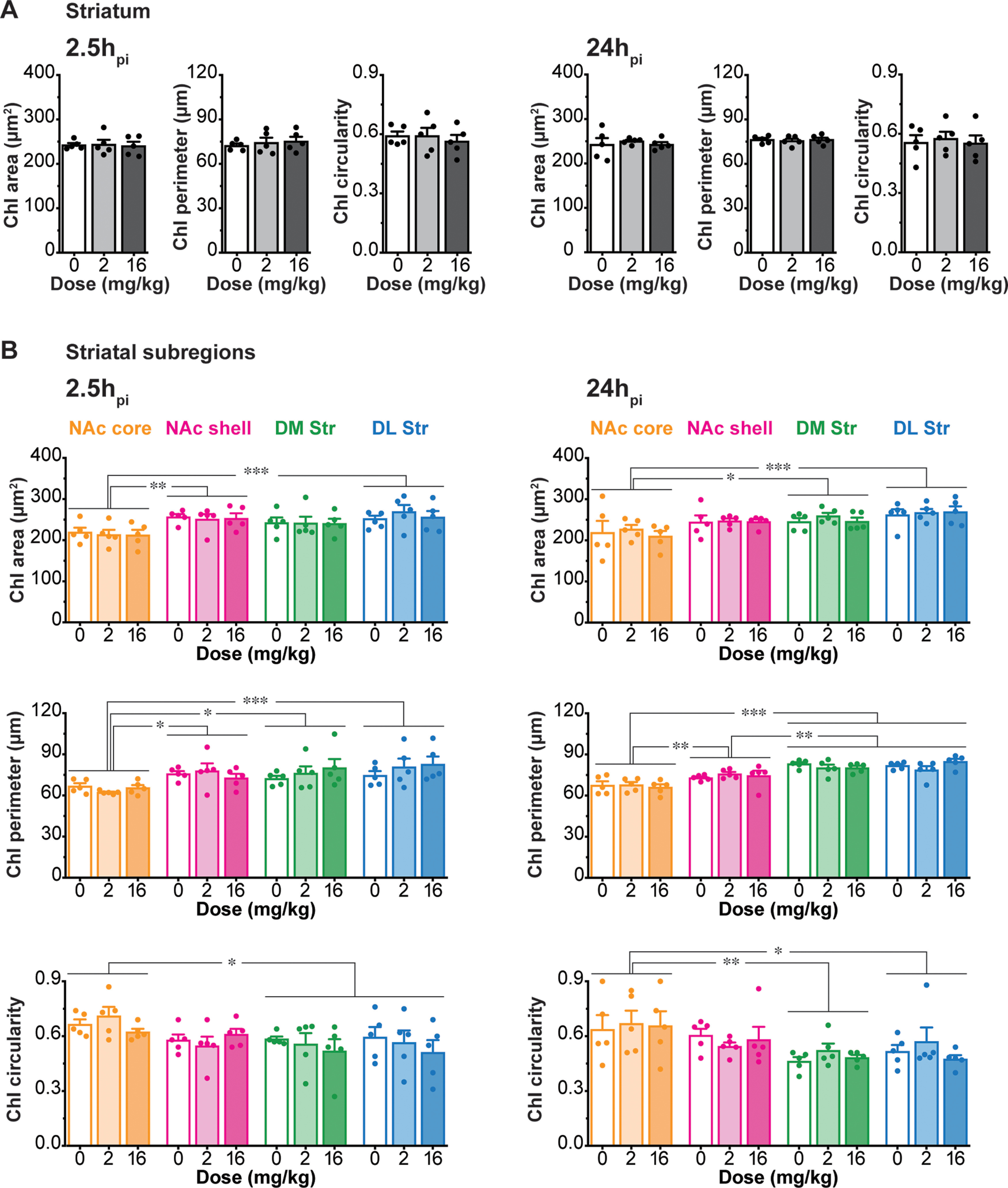
Morphology of ChIs is not affected by AMPH. ***A–B***, Morphologic characteristics of ChIs in the whole Str (***A***) and each Str subregion (***B***) in the same hemisections as shown in the previous figure: area (μm^2^), perimeter (μm), and circularity after saline (0 mg/kg), low-dose (2 mg/kg), or high-dose (16 mg/kg) AMPH, at 2.5h_pi_ (left) and 24h_pi_ (right). Dots in bar graphs show the average measurements per animal; *p < 0.05, **p < 0.01, ***p < 0.001.

We examined the shape of ChIs based on their cytoplasmic ChAT immunoreactivity. AMPH did not affect ChI soma area (2.5h_pi_: saline 242.5 ± 4.4 μm^2^, low-dose 243.6 ± 10.5 μm^2^, high-dose 240.4 ± 9.6 μm^2^; 24h_pi_: saline 242.6 ± 14.6 μm^2^, low-dose 250.1 ± 3.0 μm^2^, high-dose 242.5 ± 5.5 μm^2^; two-way ANOVA; treatment effect, *F*_(2,24)_ = 0.21, *p *=* *0.81; time effect, *F*_(1,24)_ = 0.16, *p *=* *0.69; treatment × time interaction, *F*_(2,24)_ = 0.07, *p *=* *0.94), perimeter (2.5h_pi_: saline 72.3 ± 1.4 μm, low-dose 74.3 ± 3.4 μm, high-dose 75.3 ± 3.1 μm; 24h_pi_: saline 76.3 ± 1.1 μm, low-dose 75.6 ± 1.4 μm, high-dose 76.3 ± 1.4 μm; two-way ANOVA; treatment effect, *F*_(2,24)_ = 0.23, *p *=* *0.80; time effect, *F*_(1,24)_ = 1.44, *p *=* *0.24; treatment × time interaction, *F*_(2,24)_ = 0.29, *p *=* *0.75) or circularity (2.5h_pi_: saline 0.60 ± 0.02, low-dose 0.59 ± 0.04, high-dose 0.56 ± 0.03; 24h_pi_: saline 0.56 ± 0.04, low-dose 0.58 ± 0.04, high-dose 0.55 ± 0.04; two-way ANOVA; treatment effect, *F*_(2,24)_ = 0.28, *p *=* *0.76; time effect, *F*_(1,24)_ = 0.56, *p *=* *0.46; treatment × time interaction, *F*_(2,24)_ = 0.07, *p *=* *0.93) in the whole Str, at any time points (Fig. 3*A*).

Although ChI morphology differed between Str subregions, AMPH did not affect soma area, perimeter, or circularity in any Str subregion or at the two different time points (area: three-way ANOVA; location effect, *F*_(3,96)_ = 13.17, *p *<* *0.001; treatment effect, *F*_(2,96)_ = 0.37, *p *=* *0.68; time effect, *F*_(1,96)_ = 0.31, *p *=* *0.58; location × treatment × time interaction, *F*_(6,96)_ = 0.18, *p *=* *0.98; perimeter: three-way ANOVA; location effect, *F*_(3,96)_ = 22.45, *p *<* *0.001; treatment effect, *F*_(2,96)_ = 0.38, *p *=* *0.68; time effect, *F*_(1,96)_ = 2.42, *p *=* *0.12; location × treatment × time, *F*_(6,96)_ = 0.83, *p *=* *0.55; circularity: three-way ANOVA; location effect, *F*_(3,96)_ = 7.69, *p *<* *0.001; treatment effect, *F*_(2,96)_ = 0.58, *p *=* *0.57; time effect, *F*_(1,96)_ = 1.36, *p *=* *0.25; location × treatment × time interaction, *F*_(6,96)_ = 0.35, *p *=* *0.91; Fig. 3*B*). Thus, neither low- nor high-dose AMPH affected ChI distribution or morphology, arguing against neurotoxic effects of a single dose of AMPH.

### AMPH attenuation of ChI activity *in vivo*

**Figure 4.**
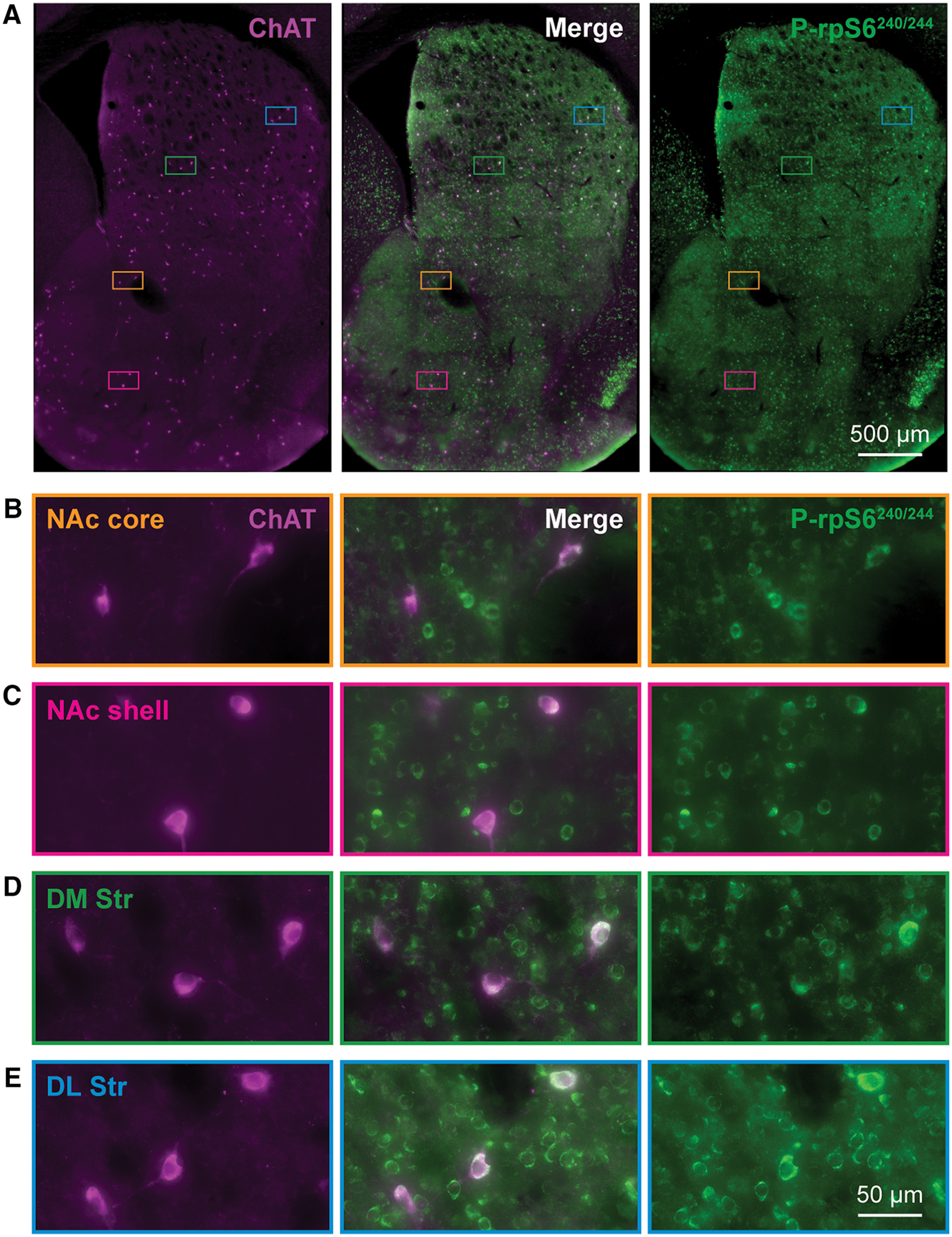
Phosphorylation of ribosomal protein S6 (p-rpS6^240/244^) in ChIs. ***A***, Low-magnification images of ChAT (purple) and p-rpS6^240/244^ (green) in a Str hemisection (bregma 0.98 mm) with merged images in the middle. Colored rectangles are representative locations of Str subregions and magnified in ***B–E***. Expanded images of the NAc core (***B***, orange), NAc shell (***C***, magenta), DM Str (***D***, green), and DL Str (***E***, blue) subregions.

**Figure 5.**
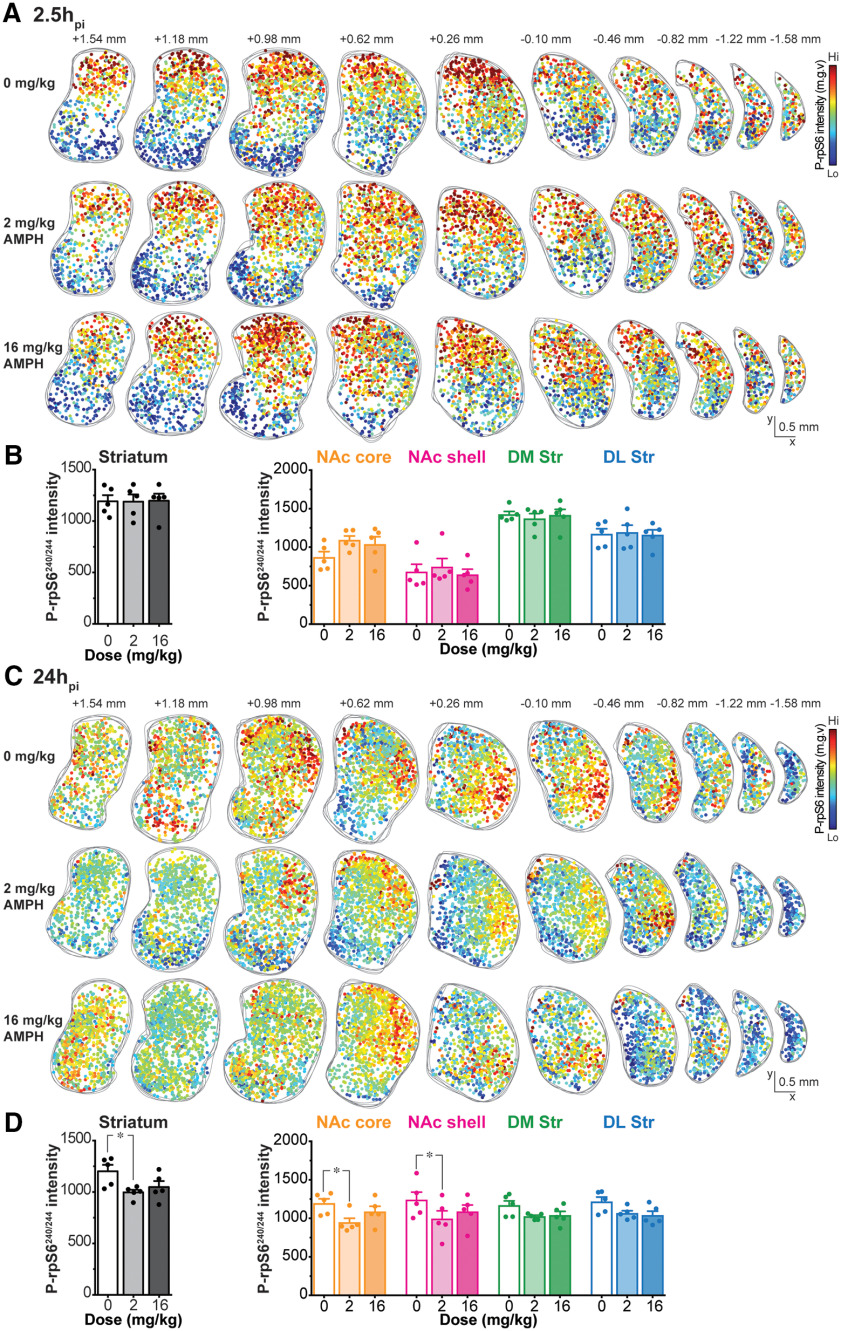
P-rpS6^240/244^ intensity in ChIs 2.5h_pi_ and 24h_pi_ AMPH. ***A***, ***C***, Spatial distribution of ChIs with relative p-rpS6^240/244^ intensity in the same 10 hemisections as shown in the previous morphology figures along the rostrocaudal axis (from bregma +1.54 to −1.58 mm), at 2.5h_pi_ (***A***) and 24h_pi_ (***C***). Spatial distribution of ChIs from five animals was superimposed for each injection group: saline (top), low-dose (middle), or high-dose (bottom) AMPH. Each dot represents one ChI and intensity of p-rpS6^240/244^ is shown on a blue (low level) to red (high level) color scale. ***B***, ***D***, left, Average p-rpS6^240/244^ intensity in ChIs in the whole Str at 2.5h_pi_ (***B***) and 24h_pi_ (***D***) after saline (0 mg/kg), low-dose (2 mg/kg), or high-dose (16 mg/kg) AMPH. Right, Box and whiskers plots showing p-rpS6^240/244^ intensity in ChIs in each Str subregion at 2.5h_pi_ (***B***) and 24h_pi_ (***D***). Dots in bar graphs show the average measurements per animal; *p < 0.05.

We mapped AMPH effects on ChI activity using p-rpS6^240/244^ as a reporter. Double immunostaining showed colocalization of ChAT and p-rpS6^240/244^ (Fig. 4). P-rpS6^240/244^ signal was also present in other Str cells, so ChAT staining was used to extract the signal specifically deriving from ChIs. We quantified p-rpS6^240/244^ intensity as the average pixel intensity in each ChAT-positive neuron, in sections from the saline, low-dose and high-dose AMPH-injected mice, at 2.5h_pi_ or 24h_pi_ (*n* = 5 animals/treatment, 10 hemisections/animal). Individual ChI locations were plotted in coronal hemisections of the Str and p-rpS6^240/244^ intensities were color-scaled (Fig. 5*A*,*C*).

At 2.5h_pi_, p-rpS6^240/244^ intensity varied among Str subregions, with higher p-rpS6^240/244^ intensity in the DM Str decreasing ventrally to the NAc shell (Fig. 5*A*). There was no apparent difference in the distribution of p-rpS6^240/244^ intensity between saline, low-dose, and high-dose AMPH-injected animals (Fig. 5*A*). Average p-rpS6^240/244^ intensity in the whole Str did not differ among saline, low-dose and high-dose AMPH-injected animals (one-way ANOVA, *F*_(2,12)_ = 0.003, *p *=* *0.99; Fig. 5*B*, left). Although average ChI p-rpS6^240/244^ intensities differed in Str subregions, neither low- nor high-dose AMPH affected ChI p-rpS6^240/244^ intensity in any Str subregion at 2.5h_pi_ (two-way ANOVA; treatment effect, *F*_(2,48)_ = 0.62, *p *=* *0.54; location effect, *F*_(3,48)_ = 41.28, *p *<* *0.001; treatment × location interaction, *F*_(6,48)_ = 0.66, *p *=* *0.68; Fig. 5*B*, right).

At 24h_pi_, ChI p-rpS6^240/244^ intensity was reduced by low-dose AMPH particularly in the NAc (Fig. 5*C*). Low-dose AMPH reduced p-rpS6^240/244^ staining more than high-dose (Fig. 5*C*). Indeed, low-dose AMPH reduced average ChI p-rpS6^240/244^ staining in the whole Str, while high-dose AMPH did not show a significant effect (one-way ANOVA, *F*_(2,12)_ = 4.35, *p *=* *0.03; Fig. 5*D*, left). Low-dose AMPH significantly reduced ChI p-rpS6^240/244^ intensity in the NAc core (*p *=* *0.043) and NAc shell (*p *=* *0.047), but not in the dorsal Str (DM Str, *p *=* *0.46; DL Str, *p *=* *0.40; Fig. 5*D*, right; two-way ANOVA; treatment effect, *F*_(2,48)_ = 8.56, *p *<* *0.001; location effect, *F*_(3,48)_ = 0.20, *p *=* *0.89; treatment × location interaction, *F*_(6,48)_ = 0.35, *p *=* *0.91). High-dose AMPH does not affect ChI p-rpS6^240/244^ intensity in any Str subregion.

**Figure 6.**
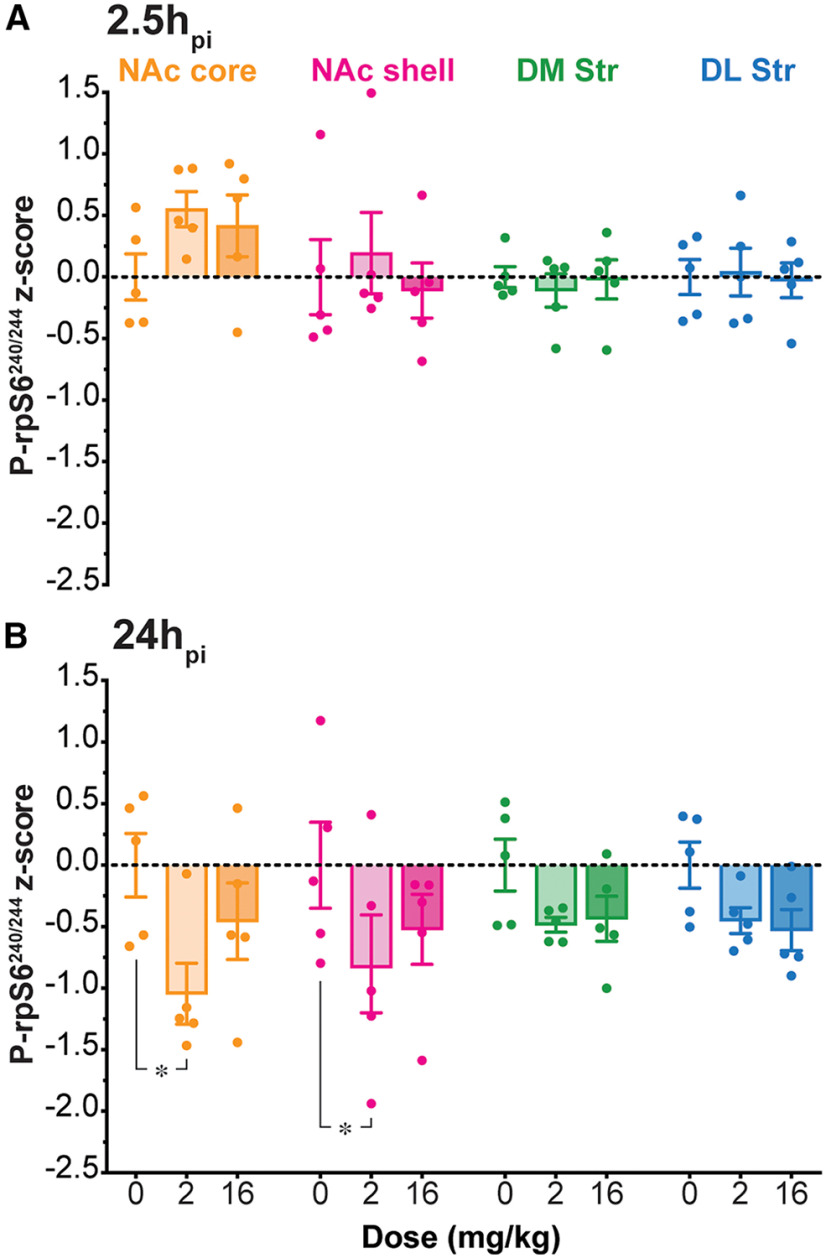
Comparison of p-rpS6^240/244^ intensity in ChIs 2.5h_pi_ and 24h_pi_ AMPH. ***A***, ***B***, Comparison of ChI p-rpS6^240/244^ intensity z-scores compared to the mean intensity in the corresponding saline-injected animals at 2.5h_pi_ (***A***) and 24h_pi_ (***B***) of saline (0 mg/kg), low-dose (2 mg/kg), or high-dose (16 mg/kg) AMPH in each Str subregion. Dots in bar graphs show the average measurements per animal; *p < 0.05.

To compare AMPH effects on p-rpS6^240/244^ intensity between the two time points, p-rpS6^240/244^ intensities in ChIs were standardized to the respective saline groups and the differences expressed as z-scores for each Str subregion. Z-scores in AMPH-injected animals at 2.5h_pi_ showed no difference from saline-injected animals in any Str subregion (two-way ANOVA; treatment effect *F*_(2,48)_ = 0.70, *p *=* *0.50; location effect *F*_(3,48)_ = 1.98, *p *=* *0.13; treatment × location interaction, *F*_(6,48)_ = 0.65, *p *=* *0.69; Fig. 6*A*). At 24h_pi_, p-rpS6^240/244^ intensity z-scores became negative after low-dose or high-dose AMPH in all Str subregions, indicating a reduction in ChI activity (two-way ANOVA; treatment effect *F*_(2,48)_ = 8.97, *p *<* *0.001; location effect *F*_(3,48)_ = 0.54, *p *=* *0.66; treatment × location interaction, *F*_(6,48)_ = 0.49, *p *=* *0.81; Fig. 6*B*). Low-dose AMPH significantly attenuated ChI p-rpS6^240/244^ intensity z-scores in the ventral subregions: NAc core (*p *=* *0.012) and shell (*p *=* *0.048), but not in the dorsal Str (DM Str, *p *=* *0.50; DL Str, *p *=* *0.60; Fig. 6*B*). AMPH effects on z-scores at 24h_pi_ were significantly different from those at 2.5h_pi_ (three-way ANOVA; time effect, *F*_(1,96)_ = 27.35, *p *<* *0.001; treatment effect, *F*_(2,96)_ = 3.18, *p *=* *0.04; location effect, *F*_(3,96)_ = 0.34, *p *=* *0.80; time × treatment × location interaction, *F*_(6,96)_ = 0.89, *p *=* *0.51).

The two doses did not affect ChI p-rpS6^240/244^ intensity in the dorsal subregions (Fig. 6*B*), nor was there any difference between the medial and lateral subregions. In the ventral subregions, the NAc core showed a similar profile of attenuation to the NAc shell. This medio-lateral concordance in the dorsal and ventral Str reinforces the differential effect in the ventral Str. The difference between the 2.5h_pi_ and 24h_pi_ cohorts reveals a time-dependent effect of a single dose of AMPH on ChI activity in the Str.

### Spontaneous firing of ChIs is not affected by AMPH

**Figure 7.**
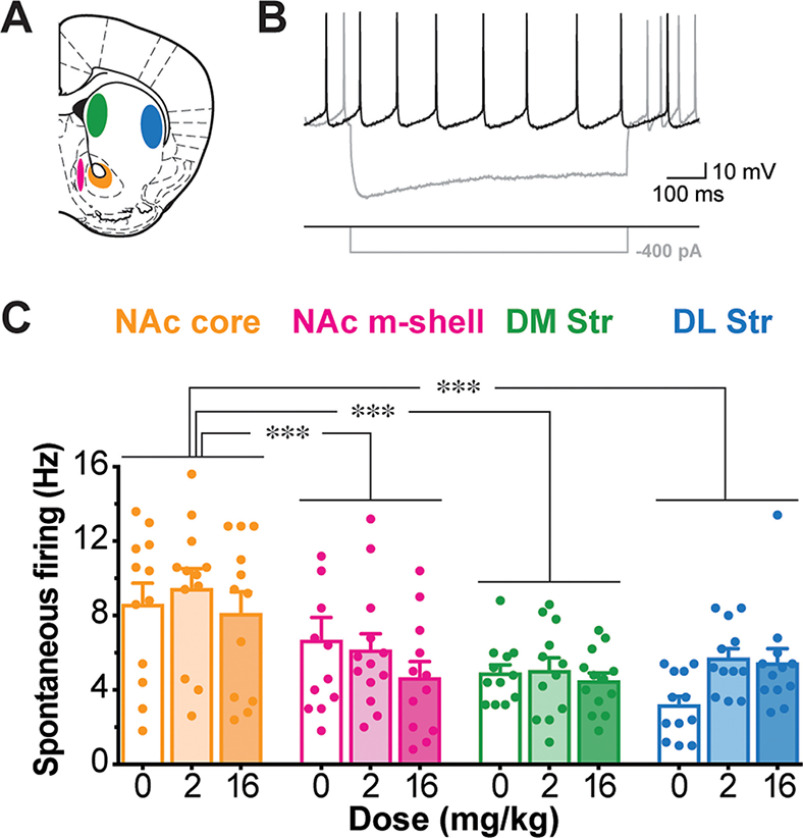
Spontaneous ChI firing 24h_pi_ AMPH. ***A***, Whole-cell recordings were made from ChIs in the four Str subregions. ***B***, An example of ChI firing recorded in the DL Str shows the characteristic spontaneous firing (black trace), and the prominent sag in response to hyperpolarizing-current injection (gray trace). ***C***, Spontaneous firing frequencies of ChIs in each Str subregion are shown after saline (0 mg/kg, *n* = 30 animals), low-dose (2 mg/kg, *n* = 22 animals), or high-dose (16 mg/kg, *n* = 28 animals) AMPH at 24h_pi_. Dots in bar graphs show measurements for individual animals; the numbers of ChIs recorded were 12–13 cells/Str subregion/treatment; ***p < 0.001.

To investigate possible mechanisms underlying the observed decrease in p-rpS6^240/244^, we recorded spontaneous firing of ChIs in slices in the four Str subregions after saline, low-dose or high-dose AMPH at 24h_pi_ (Fig. 7*A*). ChIs were identified visually by large soma size, confirmed by spontaneous firing and voltage sag in response to hyperpolarizing-current injection (Fig. 7*B*), as described previously (Chuhma et al., 2014). Although firing frequencies of ChIs varied significantly among Str subregions, AMPH did not affect firing frequencies of ChIs in any Str subregion (two-way ANOVA; treatment effect, *F*_(2,134)_ = 1.21, *p *=* *0.30; location effect, *F*_(3,134)_ = 13.30, *p *<* *0.001; treatment × location interaction, *F*_(6,134)_ = 1.12, *p *=* *0.36; Fig. 7*C*). Thus, neither low- nor high-dose AMPH affected the intrinsic firing of ChIs in the deafferented slice, at 2.5h_pi_ or 24h_pi_, suggesting AMPH effects on ChI activity are because of extra-Str synaptic input.

## Discussion

ChIs are principal targets of DA neurons and subject to regionally heterogeneous modulation. Here, we mapped the downstream effects of a single AMPH dose on ChI activity using p-rpS6^240/244^ as a ChI-preferential activity-dependent marker. The single dose of AMPH did not affect the distribution, overall morphology, or spontaneous firing of ChIs in any Str subregion, arguing against neurotoxic effects of AMPH. While AMPH had no effect on *in vivo* ChI activity at 2.5h_pi_, it significantly attenuated ChI activity at 24h_pi_ in the ventral Str/NAc. In the NAc, the attenuation in ChI activity after low-dose was greater than after high-dose. In the dorsal Str, no significant difference in ChI activity was observed after either low-dose or high-dose AMPH. Thus, a single dose of AMPH has delayed regionally heterogeneous effects on ChI activity, with a dose-dependency in the NAc.

### Distribution, morphology, and spontaneous firing of ChIs in the Str

In rodents (Gonzales and Smith, 2015), non-human primates (Brauer et al., 2000) and humans (Holt et al., 1996), the average size of ChIs in the NAc is smaller than in the dorsal Str. Here, we found that ChIs in the NAc core were significantly smaller and more elongated compared with those in other Str subregions, and that the morphology of ChIs soma (area, perimeter and circularity) differed among Str subregions. We also confirmed the differential distribution of ChIs in Str subregions (Gonzales and Smith, 2015). ChIs are denser in the NAc medial shell, as previously described in mice (Matamales et al., 2016a), rats (Phelps and Vaughn, 1986), and primates (Brauer et al., 2000).

A single injection of AMPH, either low-dose or high-dose, did not affect ChI distribution or soma morphology in any Str subregion, at either time point, showing these doses were not neurotoxic. Although AMPH neurotoxicity on DA neurons has been known for some time (Wagner et al., 1980; Ricaurte et al., 1984; Ryan et al., 1990; Miller and O’Callaghan, 1996; Krasnova et al., 2001, 2005; Granado et al., 2018), no study has focused on downstream neurotoxic effect on Str ChIs. To cause a significant toxic effect on ChIs, a higher dose of a more potent psychostimulant appears to be required; a single high-dose (30 mg/kg) of methamphetamine was found to induce a loss of 29% of ChIs in the dorsal Str (Zhu et al., 2006; Goodwin et al., 2009).

Although ChI spontaneous firing rates differed among Str subregions (Chuhma et al., 2014; Gonzales and Smith, 2015), a previous study found that a single dose of AMPH at 2.5h_pi_ did not affect intrinsic firing of ChIs in any Str subregion (Chuhma et al., 2014). Here, we have found that a single dose of AMPH at 24h_pi_, either low-dose or high-dose, did not affect the spontaneous firing of ChIs in the slice, arguing that the effects of AMPH on ChI activity, measured with p-rpS6^240/244^ at 24h_pi_, involve extrinsic synaptic input to the Str.

### Single dose of AMPH affects ChI activity

P-rpS6^240/244^ signal reports the integrated activity and p-rpS6^240/244^ intensity changes appear to be detected 60 min after pharmacological or behavioral manipulations (Bertran-Gonzalez et al., 2012), suggesting that p-rpS6^240/244^ is suitable to study ChI activity at 2.5h_pi_ or later (Knight et al., 2012). Therefore, the lack of AMPH effect at 2.5h_pi_ is not because of temporal limits of p-rpS6^240/244^ measurement. Stress increases p-rpS6^240/244^ intensity (Knight et al., 2012; Biever et al., 2015a), this may be reflected in the greater p-rpS6^240/244^ intensity in the 2.5h_pi_ compared with the 24h_pi_ saline controls.

In the present study, p-rpS6^240/244^ intensity in ChIs was not affected at 2.5h_pi_ after low-dose or high-dose AMPH, while ChI activity modulation via DA neuron glutamatergic cotransmission is dose dependently attenuated after a single dose of AMPH 2.5h_pi_ (Chuhma et al., 2014). This discrepancy could be because of differences in the measurements; p-rpS6^240/244^ reflects the tonic *in vivo* activity of ChIs, which also receive cortical and thalamic glutamatergic inputs in addition to DA neuron inputs (Lim et al., 2014), in contrast to the short phasic firing control of ChIs by DA neuron synaptic inputs.

Psychostimulants, including cocaine, methamphetamine and AMPH, are associated with an overall downregulation of DA transmission, both DA release and D2 receptor levels (Ashok et al., 2017). So, we should have expected an increase in ChI activity because of the loss of D2 receptor inhibition. In contrast, the attenuation of ChI activity at 24h_pi_ argues for polysynaptic effects extending beyond direct effects on DA neuron presynaptic terminals. Indeed, AMPH-induced DA release has an onset of minutes and lasts for about 1 h in rodents (Sulzer, 2011), in parallel with behavioral activation. Tonic attenuation of cortical or thalamic glutamatergic inputs may be caused by polysynaptic modulation, resulting in delayed attenuation of ChI activity. Since AMPH does not affect p-rpS6^240/244^ levels or protein synthesis in the Str within 2 h following injection (Rapanelli et al., 2014; Biever et al., 2015b), 2.5 h does not appear to be sufficient to cause long-term circuit changes.

Polysynaptic mechanisms that could contribute to observed decreases in ChI activity in the ventral Str/NAc may involve AMPH effects on other neurotransmitters besides DA. Glutamate efflux in the ventral tegmental area (VTA) is affected by AMPH administration, although both an increase (Xue et al., 1996) and a decrease (Wolf and Xue, 1998) of glutamate efflux have been observed. Acute AMPH exposure induces attenuation of excitatory glutamatergic synaptic transmission in the VTA by activation of serotonin receptors (Jones and Kauer, 1999). AMPH also indirectly affects DA release by stimulating the trace amine-associated receptors (TAAR1) expressed in DA neuron presynaptic terminals (Underhill et al., 2021).

### ChIs in psychostimulant-induced changes

In the present study, low-dose AMPH significantly attenuated ChI activity in the ventral Str/NAc, a crucial site of psychostimulant action (Russo et al., 2010; Sulzer, 2011). DA neurons projecting to the ventral Str/NAc that corelease glutamate (Hnasko et al., 2010; Stuber et al., 2010) can drive burst firing in ChIs (Chuhma et al., 2014; Mingote et al., 2019). A single dose of AMPH attenuates glutamate cotransmission (Chuhma et al., 2014), and mice with conditional reduction in glutamate cotransmission show an attenuated sensitization to repeated AMPH (Mingote et al., 2017). Similarly, we found here that AMPH attenuated ChI activity at 24h_pi_ only in the ventral Str/NAc, suggesting that DA neuron glutamate cotransmission may be one of the factors responsible for NAc-selective attenuation of ChIs by low-dose AMPH, in addition to attenuation of phasic firing control through direct synaptic connections of DA neurons.

Although psychostimulant addiction involves repeated use, a single dose of AMPH can induce enduring Str circuit changes, drug-dependent behavior and negative affective states, such as anhedonia, depression and anxiety (Vanderschuren et al., 1999; Koob and Le Moal, 2001; Xia et al., 2008; Kameda et al., 2011; Li et al., 2017; Jing et al., 2018; Rincón-Cortés et al., 2018; Jayanthi et al., 2020). Interestingly, even a single dose of AMPH has been found to induce behavioral and neurochemical sensitization, which appears to increase over weeks (Robinson, 1984; Vanderschuren et al., 1999). Our results, in line with these previous findings, point to the relevance of a single dose of AMPH for elucidating drug-induced plasticity. Enduring alterations in ChI activity following acute AMPH exposure point to ChIs as a key component of drug-induced plasticity in the Str circuitry. Further studies using mice with restricted expression of opsins in ChAT neurons will be required to explore whether this reduction in NAc ChI activity is important in subsequent drug-dependent behavior.

## Synthesis

Reviewing Editor: Lindsay De Biase, University of California, Los Angeles Decisions are customarily a result of the Reviewing Editor and the peer reviewers coming together and discussing their recommendations until a consensus is reached. When revisions are invited, a fact-based synthesis statement explaining their decision and outlining what is needed to prepare a revision will be listed below. The following reviewer(s) agreed to reveal their identity: NONE. Note: If this manuscript was transferred from JNeurosci and a decision was made to accept the manuscript without peer review, a brief statement to this effect will instead be what is listed below.

The present study is aimed at understanding the acute and enduring effects of a single exposure to amphetamine on cholinergic neuron activity in various striatal sub-regions. The authors first demonstrate that amphetamine administration does not change cholinergic neuron density or morphology (as assessed by ChAT-immunoreactivity) across striatal sub-regions. The authors subsequently demonstrate that striatal cholinergic neuron activity, as assessed by p-rpS6 immunostaining, is decreased in the whole striatum at 24 post amphetamine for the low, but not high dose, of amphetamine. Overall, the data are well presented, and the analysis of this data set is rigorous and transparent. A major strength of this manuscript is the broad neuroanatomical survey. However, the study is correlational in nature. Lack of causal experiments and lack of endpoints beyond p-rpS6 are a major limitation of the advancement in the present study. Such experiments would increase the significance of the findings, but are explicitly requested given limitations imposed by the pandemic.

Major concerns:

1. Based on the reported degrees of freedom, it appears that authors sometimes use individual neurons as the number of observations. Since these experiments were designed to test effects of amphetamine, which was given per animal, the statistics should be run using animals and not neurons to determine degrees of freedom. I would expect variability at the animal level in terms of reactivity to amphetamine and quality of perfusion that would introduce variance across all data from that individual.

2. There appeared to be large variability within group relative to the difference in mean values between groups. And there were relatively small group sizes (n=5) compared to the multiple (4-5?) p-rpS6 related measurements collected per experimental subject. (How many statistical comparisons is each vehicle-injected mouse used in?) In this light, authors need to more convincingly establish that the regional heterogeneity or dose-dependency are not simply false discoveries.

3. Both reviewers note the correlational nature of the data and the lack of causal experiments linking behavior and histological observations. For example, experiments delineating whether rescuing AMPH-induced reductions in ChI activity impact the behavioral consequences of AMPH would greatly increase the impact of the study. If the authors are not in a position to add such behavioral experiments, reviewers suggest several alternatives that will also extend the impact of the manuscript. 1) The authors should also examine the rpS6 signals in non-ChI neurons (presumably MSNs). Are there key relationships between local patterns of MSN activity, as reported by rpS6, and observed changes in ChI activity? Similarly, can the authors estimate local MSNs that are likely to be within the “territory” of a given ChI to look for relationships between rpS6 signals in these ChIs and rpS6 signals in nearby MSNs? 2) There is a “hot spot” in the dorsal rostromedial shell with unique properties, which would be interesting to look at in this data. Is there anything distinct about patterns of MSN or ChI rpS6 signals in this hot spot region?

Minor Concerns:

1. The authors have not adequately explained the potential physiological significance of the rpS6 measurements. What do negative results @ 2.5 hours mean? Does acute amphetamine have no effect on cholinergic firing rates? Is this conclusion supported/refuted by literature? What do positive results @ 24 hours mean? Is this a delayed molecular response to the acute effects of drug? Or does it reflect some long-lasting process that may account for subsequent differences in drug-associated behavior? Psychomotor sensitization is commonly observed to gradually develop over repeated administration of psychostimulant drugs. Even after a single administration, sensitization is strongest several weeks afterwards (eg, Vandenshuren 1999; Shuster 1977). Is this a consequence of dopamine depletion, either directly or indirectly (e.g. through reduced levels of behavioral activity). Thus would we expect to see the effect disappear at later time points (at which sensitization would be more evident)? Is it a consequence of toxicity in a manner that is not (yet) evident by immunohistochemical measures used here?

2. The authors should explain that DAT-Cre animals were used but the presence of Cre is not essential for the study. They should also provide an explanation that this mouse line has decrease DAT expression and should briefly discuss this as a caveat.

3. The data on ChAT neuron cell size is based on ChAT immunoreactivity not a cell-filling stain or fluorophore. The authors should be more conservative in stating that this represents area occupied by ChAT-immunoreactivity and not somal size or shape.

4. In the first paragraph of the discussion, the authors claim that p-rpS6 is used as a selective marker for ChI activity. This is not strictly accurate since MSNs also display changes in p-rpS6, which is quite evident in their representative images.

5. The authors allude to polysynaptic effects that may underlie the effects of amphetamine at 24 hr post injection. However, amphetamine has other targets beyond DA terminals that regulate its actions including increasing glutamatergic transmission and effects on TAARs. The authors should discuss this as alternative explanations.

6. Sexes - n per group and were there sex effects seen?

7. Graphical annotations of statistical significance do not appear correct. For example in Fig 6B, NAc shell 2 vs 16 mg/kg appear to be marked as different at the p=0.01 level. This cannot possibly be correct. Am I misinterpreting the brackets?

## Author Response

Thank you for reviewing our manuscript and for the helpful comments. Please find here our point-to-point replies. In the following, reviewers’ comments are indicated in black, and our replies in blue text, followed by the revised text in the manuscript highlighted in yellow.

Major concerns:

1. Based on the reported degrees of freedom, it appears that authors sometimes use individual neurons as the number of observations. Since these experiments were designed to test effects of amphetamine, which was given per animal, the statistics should be run using animals and not neurons to determine degrees of freedom. I would expect variability at the animal level in terms of reactivity to amphetamine and quality of perfusion that would introduce variance across all data from that individual.

Agreed. All statistical analyses are now run per animal. The following changes have been made in the Results, and Figures 6 and 7, where dots in bar graphs now show the average measurements per animal:

Although average ChI p-rpS6240/244 intensities differed in Str subregions, neither low- nor high- dose AMPH affected ChI p-rpS6240/244 intensity in any Str subregion at 2.5hpi (two-way ANOVA; treatment effect, F(2, 48) = 0.62, p = 0.54; location effect, F(3, 48) = 41.28, p < 0.001; treatment × location interaction, F(6, 48) = 0.66, p = 0.68) (Fig. 6B, right).

Low-dose AMPH significantly reduced ChI p-rpS6240/244 intensity in the NAc core (p = 0.043) and

NAc shell (p = 0.047), but not in the dorsal Str (DM Str, p = 0.46; DL Str, p = 0.40)(Fig.6D, right)

(two-way ANOVA; treatment effect, F(2, 48) = 8.56, p < 0.001; location effect, F(3, 48) = 0.20, p = 0.89;

treatment × location interaction, F(6, 48) = 0.35, p = 0.91). High-dose AMPH does not affect ChI p-

rpS6240/244 intensity in any Str subregion.

Z-scores in AMPH-injected animals at 2.5hpi did not differ significantly from saline-injected animals in any Str subregion (Fig. 7A) (two-way ANOVA; treatment effect F(2, 48) = 0.70, p = 0.50; location effect F(3, 48) = 1.98, p = 0.13; treatment × location interaction, F(6, 48) = 0.65, p = 0.69).

At 24hpi, p-rpS6240/244 intensity z-scores became negative after low- or high-dose AMPH in all Str subregions, indicating a reduction in ChI activity (Fig. 7B) (two-way ANOVA; treatment effect F(2,

48) = 8.97, p < 0.001; location effect F(3, 48) = 0.54, p = 0.66; treatment × location interaction, F(6, 48)

= 0.49, p = 0.81).

Low-dose AMPH significantly attenuated ChI p-rpS6240/244 intensity z-scores in the ventral subregions: NAc core (p = 0.012) and shell (p = 0.048), but not in the dorsal Str (DM Str, p = 0.50; DL Str, p = 0.60) (Fig. 7B).

2. There appeared to be large variability within group relative to the difference in mean values between groups. And there were relatively small group sizes (n=5) compared to the multiple (4-

5?) p-rpS6 related measurements collected per experimental subject. (How many statistical comparisons is each vehicle-injected mouse used in?) In this light, authors need to more convincingly establish that the regional heterogeneity or dose-dependency are not simply false discoveries.

In our study, the same cohorts of animals and the same sections were analyzed for ChAT immunostaining (morphology of ChIs) or p-rps6 (activity of ChIs). We clarified this in the figure legends and Results:

Figure 2: Group n’s are given in Figure 1.

Figure 3: Morphological characteristics of ChIs in the whole Str (A) and each Str subregion (B) in the same hemisections as shown in the previous figure.

Figure 6: Spatial distribution of ChIs with relative p-rpS6240/244 intensity in the same 10 hemisections as shown in the previous figures along the rostrocaudal axis (from bregma +1.54 mm to -1.58 mm), at 2.5hpi (A) and 24hpi (C).

We quantified p-rpS6240/244 intensity as the average pixel intensity in each ChAT positive neuron, in sections from the same saline, low- and high-dose AMPH-injected mice, at 2.5hpi or 24hpi (n =

5 animals/treatment, 10 hemisections/animal).

P-rpS6 related measurements were not compared multiple times. ChI anatomical measurement (Figures 2 and 3) is different from p-rpS6 measurement. Those are different data sets (from the same sections) using only ChAT-positive neurons images to examine the distribution (count, density) and morphology (area, perimeter, circularity) of ChIs. P-rpS6 related measurements using the P-rps6 image were only used once per group (Figure 6). Z-scores (which look like overlapped measurements) were just for standardization to allow comparisons between 2.5hpi and 24hpi experiments (Figure 7). Therefore, regional heterogeneity observed here cannot be false positive since no multiple repeated comparison was done here. Regarding the group size, the power analysis is included in the Materials and Methods:

Sample size were determined based on estimated effect sizes using G*Power 3.1 (G*Power, RRID: SCR_013726), setting α = 0.05 and power = 0.8 (Cunningham and McCrum-Gardner, 2007; Faul et al., 2007). For the immunocytochemistry experiments, we predicted an effect size Cohen’s D = 0.97, which resulted in an estimated sample size of 5 per group. For the electrophysiological experiments, we predicted an effect size Cohen’s D = 0.32, which resulted in an estimated sample size of 12 per group.

3. Both reviewers note the correlational nature of the data and the lack of causal experiments linking behavior and histological observations. For example, experiments delineating whether rescuing AMPH-induced reductions in ChI activity impact the behavioral consequences of AMPH would greatly increase the impact of the study.

The present study was designed to evaluate dose-dependent effects of AMPH on ChI activity, for which behavior was a gatekeeper. We clarified it in the revised manuscript (see Results):

Behavioral observations were used as a gatekeeper to confirm the dose-dependent effects of

AMPH.

If the authors are not in a position to add such behavioral experiments, reviewers suggest several alternatives that will also extend the impact of the manuscript. 1) The authors should also examine the rpS6 signals in non-ChI neurons (presumably MSNs). Are there key relationships between local patterns of MSN activity, as reported by rpS6, and observed changes in ChI activity? Similarly, can the authors estimate local MSNs that are likely to be within the “territory"

of a given ChI to look for relationships between rpS6 signals in these ChIs and rpS6 signals in nearby MSNs?

The phosphorylation of rpS6 occurs sequentially at 5 serine residues: 236, 235, 240, 244 and 247. When comparing the levels of phosphorylation in two different pairs of C-terminal serines (p- rpS6235/236 and p-rpS6240/244), Bertran-Gonzalez group showed a clear p-rpS6240/244 signal preferentially expressed in ChIs (Bertran-Gonzalez et al., 2012). Regulation of p-rpS6 described in the literature in the two populations of striatal projection MSNs occurs at serine 235 and 236 residues (Valjent et al., 2011; Biever et al., 2015). The experiments presented in our study were done using only the antibody anti-p-rpS6240/244 which will not allow us to compare ChIs and MSNs pattern of activity. To avoid any misunderstanding, we replaced ’p-rpS6’ by ’p-rpS6240/244’ throughout the manuscript. We also added in Introduction:

Bertran-Gonzalez et al. showed a clear p-rpS6240/244 signal preferentially expressed in ChIs, in contrast to a much weaker signal of p-rpS6235/236 (Bertran-Gonzalez et al., 2012).

We have added electrophysiological recordings of cholinergic interneuron firing in the four striatal subregions, including the nucleus accumbens medial shell hotspot, in the Materials and Methods, Results, Figure 4, and Discussion:

Slice electrophysiology and Analysis

For electrophysiology recording, mice were anesthetized with ketamine (90 mg/kg)/xylazine (7 mg/kg). After confirmation of full anesthesia, mice were decapitated and brains quickly removed in ice-cold high-glucose artificial cerebrospinal fluid (ACSF) (in mM: 75 NaCl, 2.5 KCl, 26 NaHCO3,

1.25 NaH2PO4, 0.7 CaCl2, 2 MgCl2 and 100 glucose, pH 7.4) saturated with carbogen (95% O2 + 5% CO2). Coronal sections of the striatum were cut, 300 mm thick, with a vibrating microtome (VT1200S, Leica), incubated in high-glucose ACSF at room temperature for at least 1 hour for recovery, then transferred to the recording chamber (submerged, 500 ml volume) on the stage of an upright microscope (BX61WI, Olympus), continuously perfused with standard ACSF (in mM:

125 NaCl, 2.5 KCl, 25 NaHCO3, 1.25 NaH2PO4, 2 CaCl2, 1 MgCl2 and 25 glucose, pH 7.4) saturated with carbogen. Recorded neurons were visualized using enhanced visible light differential

interference contrast (DIC) optics with a scientific c-MOS camera (ORCA-Flash4.0LT, Hamamatsu Photonics). ChIs were identified visually by large soma size, confirmed by spontaneous firing, shallow resting membrane potentials (around 60 mV) and voltage sag by 400 pA current injection (700 msec duration) (Chuhma et al., 2014; Chuhma et al., 2018). Recording patch pipettes were fabricated from standard-wall borosilicate glass capillary with filament (World Precision Instruments). Pipette resistance was 4-9 MΩ and series resistance was 7-32 MΩ. Composition of intracellular solution was (in mM): 135 K+-methane sulfonate (MeSO4), 5 KCl, 2 MgCl2, 0.1 CaCl2,

10 HEPES, 1 EGTA, 2 ATP and 0.1 GTP, pH 7.25. Recording was done with an Axopatch 200B amplifier (Molecular Devices) in fast current clamp mode. All recordings were done at 32-34{degree sign}C (TC 344B Temperature Controller, Warner Instruments). No more than 4 cells were recorded per animal. Data were filtered at 5 kHz using a 4-pole Bessel filter, digitized at 5 kHz (Digidata 1550A, Molecular Devices) and recorded using pClamp 10 (Molecular Devices; RRID:SCR_011323). Electrophysiological data were analyzed with Axograph X (Axograph Science; RRID:SCR_014284). Firing frequencies were calculated as average frequency in a 2 s window obtained from ten consecutive traces.

Spontaneous firing of ChIs is not affected by AMPH

We then addressed AMPH effects on the spontaneous firing of ChIs. Previous work showed that AMPH at 2.5hpi did not affect ChI firing (Chuhma et al., 2014). However, the effect of AMPH at the 24hpi time point was not examined. To address this, we recorded spontaneous firing of ChIs in slices in the four Str subregions after saline, low- or high-dose AMPH at 24hpi (Fig. 4A). ChIs were identified visually by large soma size, confirmed by spontaneous firing and voltage sag by

400 pA current injection (Fig. 4B), as described previously (Chuhma et al., 2014). Although firing frequencies of ChIs varied significantly among Str subregions, AMPH did not affect firing frequencies of ChIs in any Str subregion (two-way ANOVA; treatment effect, F(2, 134) = 1.21, p =

0.30; location effect, F(3, 134) = 13.30, p < 0.001; treatment × location interaction, F(6, 134) = 1.12, p

= 0.36) (Fig. 4C). Thus, neither low- nor high-dose AMPH affected the intrinsic firing of ChIs in the deafferented slice, at 2.5hpi or 24hpi.

Figure 4. Spontaneous ChI firing 24hpi AMPH

(A) Whole cell recordings were made from ChIs in the four Str subregions. (B) An example of ChI firing recorded in the DL Str shows the characteristic spontaneous firing (black trace), and the prominent sag in response to hyperpolarizing-current injection (gray trace). (C) Spontaneous firing frequencies of ChIs in each Str subregion are shown after saline (0 mg/kg, n = 30 animals), low-dose (2 mg/kg, n = 22 animals) or high-dose (16 mg/kg, n = 28 animals) AMPH at 24hpi. Dots in bar graphs show measurements for individual animals; the numbers of ChIs recorded were 12-

13 cells/Str subregion/treatment. *** indicate p < 0.001.

Although ChI spontaneous firing rates differed among Str subregions (Chuhma et al., 2014; Gonzales and Smith, 2015), a previous study found that a single dose of AMPH at 2.5hpi did not affect intrinsic firing of ChIs in any Str subregion (Chuhma et al., 2014). Here, we have found that a single dose of AMPH at 24hpi, either low- or high-dose, did not affect the spontaneous firing of ChIs in the slice, arguing that the effects of AMPH on ChI activity, measured with p-rpS6 at 24hpi, involve extrinsic synaptic input to the Str.

2) There is a “hot spot” in the dorsal rostromedial shell with unique properties, which would be interesting to look at in this data. Is there anything distinct about patterns of MSN or ChI rpS6 signals in this hot spot region?

We cannot reply to this interrogation as the striatal subregions delineations done in our study included both nucleus accumbens medial and lateral shell. When we look at the spatial distribution, in the saline-injected mice of the 2.5hpi cohort, we observed a lower ChI p-rpS6 signal in the nucleus accumbens medial shell in comparison to dorsal subregions. This was supported by literature (Matamales et al., 2016). However, in the saline-injected mice of the 24hpi cohort, this is less clear.

Minor Concerns:

1. The authors have not adequately explained the potential physiological significance of the rpS6 measurements.

This is added now in the Results:

P-rpS6240/244 signal reports the integrated activity of ChIs.

What do negative results @ 2.5 hours mean? What do positive results @ 24 hours mean? Is this a delayed molecular response to the acute effects of drug? Or does it reflect some long-lasting process that may account for subsequent differences in drug-associated behavior?

In regards to the significance of negative results at 2.5hpi and positive results at 24hpi, we first discussed that the lack of AMPH effect could not be due to a delayed p-rpS6240/244 response, since the signal can be detected 60 min after pharmacological manipulations. We could have observed at 2.5hpi: (1) an attenuation of ChI activity, due to AMPH attenuation of DA neuron glutamatergic cotransmission (Chuhma et al., 2014) or (2) an increase of ChI activity due to the loss of D2 receptor inhibition (downregulation of DA transmission reported in clinical studies). However, that is not the case here. In addition, previous work in our laboratory showed that acute AMPH at 2.5hpi did not affect spontaneous firing of ChIs. Our electrophysiology data added in this revised manuscript show that acute AMPH at 24hpi also did not affect spontaneous firing of ChIs. This strengthens our hypothesis that AMPH effects on ChI activity are most likely synaptic. Presumably, 2.5h is not sufficient to engender longer-term circuit changes involving ChIs as seen at 24h to integrate all the synaptic inputs leading to the attenuation observed in the p-rpS6240/244 signal (see Discussion).

P-rpS6240/244 signal reports the integrated activity and p-rpS6240/244 intensity change appears to be detected 60 min after pharmacological or behavioral manipulations (Bertran-Gonzalez et al.,

2012), suggesting that p-rpS6240/244 is suitable to study ChI activity at 2.5hpi or later (Knight et al.,

2012). Therefore, the lack of AMPH effect at 2.5hpi is not due to temporal limits of p-rpS6240/244

measurement. Stress increases p-rpS6240/244 intensity (Knight et al., 2012; Biever et al., 2015); this may be reflected in the greater p-rpS6240/244 intensity in the 2.5hpi compared to the 24hpi saline

controls.

The present results confirm the dorsoventral gradient in ChI activity (Matamales et al., 2016). In the present study, p-rpS6240/244 intensity in ChIs was not affected at 2.5hpi after low- or high-dose AMPH, while ChI activity modulation via DA neuron glutamatergic cotransmission is dose- dependently attenuated after a single dose of AMPH 2.5hpi (Chuhma et al., 2014). This discrepancy could be due to differences in the measurements; p-rpS6240/244 reflects the tonic in vivo activity of ChIs, which also receive cortical and thalamic glutamatergic inputs in addition to DA neuron inputs (Lim et al., 2014), in contrast to the short phasic firing control of ChIs by DA neuron synaptic inputs.

Psychostimulants, including cocaine, methamphetamine and AMPH, are associated with an overall downregulation of DA transmission, both DA release and D2 receptor levels (Ashok et al.,

2017). So, we should have expected an increase in ChI activity due to the loss of D2 receptor inhibition. In contrast, the attenuation of ChI activity at 24hpi argues for polysynaptic effects extending beyond direct effects on DA neuron presynaptic terminals. Indeed, AMPH-induced DA release has an onset of minutes and lasts for about an hour in rodents (Sulzer, 2011), in parallel with behavioral activation. Tonic attenuation of cortical or thalamic glutamatergic inputs may be caused by polysynaptic modulation, resulting in delayed attenuation of ChI activity. Since AMPH does not affect p-rpS6240/244 levels or protein synthesis in the Str within 2 hours following injection (Rapanelli et al., 2014; Biever et al., 2015), 2.5 hours does not appear to be sufficient to cause long-term circuit changes.

Does acute amphetamine have no effect on cholinergic firing rates? Is this conclusion supported/refuted by literature?

See response to Reviewer comment #3 (electrophysiological recordings).

Our findings are in line with previous studies that showed no AMPH effect on spontaneous firing of ChIs in any striatal subregion 2.5h post-injection (Chuhma et al., 2014). In contrast, cocaine has been shown to increase the spontaneous activity of ChIs (Witten et al., 2010).

Psychomotor sensitization is commonly observed to gradually develop over repeated administration of psychostimulant drugs. Even after a single administration, sensitization is strongest several weeks afterwards (eg, Vandenshuren 1999; Shuster 1977). Is this a consequence of dopamine depletion, either directly or indirectly (e.g. through reduced levels of behavioral activity). Thus would we expect to see the effect disappear at later time points (at which sensitization would be more evident)?

Amphetamine-induced behavioral sensitization gradually developed even after only a single AMPH administration, with sensitization being most pronounced at 3 weeks after treatment (Vanderschuren et al., 1999). We added now in the Discussion:

Interestingly, even a single dose of AMPH has been found to induce behavioral and neurochemical sensitization, which appears to increase over weeks (Robinson, 1984; Vanderschuren et al., 1999).

Is it a consequence of toxicity in a manner that is not (yet) evident by immunohistochemical measures used here?

In all parameters, we verified for the distribution (count, density) and morphology (area, perimeter, circularity) of ChIs, none were significantly altered by acute AMPH. This is supported by the literature, where only a high dose of 30 mg/kg has been found to be toxic (Zhu et al.,

2006). Therefore, we argue against toxic effect of acute AMPH at the dose of 2 or 16 mg/kg (see

Results and Discussion).

2. The authors should explain that DAT-Cre animals were used but the presence of Cre is not essential for the study. They should also provide an explanation that this mouse line has decrease DAT expression and should briefly discuss this as a caveat.

This is added now to the Materials and Methods:

The DAT-IRES-Cre/+;ROSA26-flox-STOP-CAG-ChR2-YFP double mutant strain (Jackson Laboratories, RRID:IMSR_JAX:006660, RRID:IMSR_JAX:024109) were used, with the same genotype as previous studies (Chuhma et al., 2014; Mingote et al., 2015; Mingote et al., 2017; Chuhma et al., 2018). The presence of Cre is not essential for the present study; the IRES-cre transgene insertion in the DA transporter (DAT) locus modestly reduces DAT expression and AMPH responsiveness (Backman et al., 2006; Chohan et al., 2020).

3. The data on ChAT neuron cell size is based on ChAT immunoreactivity not a cell-filling stain or fluorophore. The authors should be more conservative in stating that this represents area occupied by ChAT-immunoreactivity and not soma size or shape.

ChAT is a cytoplasmic enzyme and so provides the equivalent of a dye fill. We have filled ChIs with Alexa 594-conjugated biocytin and shown perfect overlap with ChAT immunostaining (n =

17 cells) (Chuhma et al., 2014, supplemental information, Figure S2). See also: Martel AC, Elseedy H, Lavigne M, Scapula J, Ghestem A, Kremer EJ, Esclapez M, Apicella P. Targeted Transgene Expression in Cholinergic Interneurons in the Monkey Striatum Using Canine Adenovirus Serotype

2 Vectors. Front Mol Neurosci 13:76, 2020. To clarify, we added to the Results:

We examined the shape of ChIs based on their cytoplasmic ChAT immunoreactivity.

4. In the first paragraph of the discussion, the authors claim that p-rpS6 is used as a selective marker for ChI activity. This is not strictly accurate since MSNs also display changes in p-rpS6, which is quite evident in their representative images.

As explained above, when comparing the levels of phosphorylation in two different pairs of C- terminal serines (p-rpS6235/236 and p-rpS6240/244), Bertran-Gonzalez et al. showed that the p- rpS6240/244 signal is preferentially expressed in ChIs (Bertran-Gonzalez et al., 2012). In the revised manuscript, we replaced the term ’selective’ by ’preferential’. We also replaced ’p-rpS6’ by ’p- rpS6240/244’ throughout the manuscript.

5. The authors allude to polysynaptic effects that may underlie the effects of amphetamine at 24 hr post injection. However, amphetamine has other targets beyond DA terminals that regulate its actions including increasing glutamatergic transmission and effects on TAARs. The authors should discuss this as alternative explanations.

This is added now in the revised manuscript (see Discussion):

AMPH affects other neurotransmitters. Glutamate efflux in the ventral tegmental area (VTA) is affected by AMPH administration, although both an increase (Xue et al., 1996) and a decrease (Wolf and Xue, 1998) of glutamate efflux have been observed. Acute AMPH exposure induces attenuation of excitatory glutamatergic synaptic transmission in the VTA by activation of serotonin receptors (Jones and Kauer, 1999). AMPH also indirectly affects DA release by stimulating the trace amine-associated receptors (TAAR1) expressed in DA neuron presynaptic terminals (Underhill et al., 2021).

6. Sexes - n per group and were there sex effects seen? This is added now in the Materials and Methods:

For the immunocytochemistry experiments, 30 mice were used at postnatal day (P) P56-82, divided in two cohorts of 15 for 2.5hpi and 15 for 24hpi. Cohorts were balanced for sex: 16 male (2.5hpi cohort: saline, n = 3; low-dose AMPH, n = 3; high-dose AMPH, n = 3 and 24hpi cohort: saline, n = 2; low-dose AMPH, n = 2; high-dose AMPH, n = 3) and 14 female (2.5hpi cohort: saline, n = 2; low-dose AMPH, n = 2; high-dose AMPH, n = 2 and 24hpi cohort: saline, n = 3; low-dose AMPH, n = 3; high-dose AMPH, n = 2) mice. For the electrophysiological experiments, 40 male (saline, n = 17; low-dose AMPH, n = 11; high-dose AMPH, n = 12) and 40 female (saline, n = 13; low-dose AMPH, n = 11; high-dose AMPH, n = 16) mice at P52-72 were used. No sex differences were observed, so data from male and female mice in each group were combined.

7. Graphical annotations of statistical significance do not appear correct. For example in Fig 6B, NAc shell 2 vs 16 mg/kg appear to be marked as different at the p=0.01 level. This cannot possibly be correct. Am I misinterpreting the brackets?

We corrected this. Statistical analyses are now run per animal (see response to Reviewer comment #1).

## References

[B1] Abudukeyoumu N, Hernandez-Flores T, Garcia-Munoz M, Arbuthnott GW (2019) Cholinergic modulation of striatal microcircuits. Eur J Neurosci 49:604–622. 10.1111/ejn.13949 29797362 PMC6587740

[B2] Ashok AH, Mizuno Y, Volkow ND, Howes OD (2017) Association of stimulant use with dopaminergic alterations in users of cocaine, amphetamine, or methamphetamine: a systematic review and meta-analysis. JAMA Psychiatry 74:511–519. 10.1001/jamapsychiatry.2017.0135 28297025 PMC5419581

[B3] Atallah HE, McCool AD, Howe MW, Graybiel AM (2014) Neurons in the ventral striatum exhibit cell-type-specific representations of outcome during learning. Neuron 82:1145–1156. 10.1016/j.neuron.2014.04.021 24908491 PMC4108162

[B4] Bäckman CM, Malik N, Zhang Y, Shan L, Grinberg A, Hoffer BJ, Westphal H, Tomac AC (2006) Characterization of a mouse strain expressing Cre recombinase from the 3' untranslated region of the dopamine transporter locus. Genesis 44:383–390. 10.1002/dvg.20228 16865686

[B5] Bertran-Gonzalez J, Chieng BC, Laurent V, Valjent E, Balleine BW (2012) Striatal cholinergic interneurons display activity-related phosphorylation of ribosomal protein S6. PLoS One 7:e53195. 10.1371/journal.pone.0053195 23285266 PMC3532298

[B6] Biever A, Valjent E, Puighermanal E (2015a) Ribosomal protein S6 phosphorylation in the nervous system: from regulation to function. Front Mol Neurosci 8:75. 10.3389/fnmol.2015.00075 26733799 PMC4679984

[B7] Biever A, Puighermanal E, Nishi A, David A, Panciatici C, Longueville S, Xirodimas D, Gangarossa G, Meyuhas O, Hervé D, Girault JA, Valjent E (2015b) PKA-dependent phosphorylation of ribosomal protein S6 does not correlate with translation efficiency in striatonigral and striatopallidal medium-sized spiny neurons. J Neurosci 35:4113–4130. 10.1523/JNEUROSCI.3288-14.2015 25762659 PMC6605295

[B8] Brauer K, Häusser M, Hartig W, Arendt T (2000) The core-shell dichotomy of nucleus accumbens in the rhesus monkey as revealed by double-immunofluorescence and morphology of cholinergic interneurons. Brain Res 858:151–162. 10.1016/s0006-8993(00)01938-7 10700608

[B9] Chohan MO, Esses S, Haft J, Ahmari SE, Veenstra-VanderWeele J (2020) Altered baseline and amphetamine-mediated behavioral profiles in dopamine transporter Cre (DAT-Ires-Cre) mice compared to tyrosine hydroxylase Cre (TH-Cre) mice. Psychopharmacology (Berl) 237:3553–3568. 10.1007/s00213-020-05635-4 32778904 PMC10120402

[B10] Chuhma N, Mingote S, Moore H, Rayport S (2014) Dopamine neurons control striatal cholinergic neurons via regionally heterogeneous dopamine and glutamate signaling. Neuron 81:901–912. 10.1016/j.neuron.2013.12.027 24559678 PMC3933825

[B11] Chuhma N, Mingote S, Yetnikoff L, Kalmbach A, Ma T, Ztaou S, Sienna AC, Tepler S, Poulin JF, Ansorge M, Awatramani R, Kang UJ, Rayport S (2018) Dopamine neuron glutamate cotransmission evokes a delayed excitation in lateral dorsal striatal cholinergic interneurons. Elife 7:e39786. 10.7554/eLife.39786 30295607 PMC6175576

[B12] Cunningham J, McCrum-Gardner E (2007) Power, effect and sample size using GPower: practical issues for researchers and members of research ethics committees. Evidence Based Midwifery 5:132–136.

[B13] Faul F, Erdfelder E, Lang AG, Buchner A (2007) G*Power 3: a flexible statistical power analysis program for the social, behavioral, and biomedical sciences. Behav Res Methods 39:175–191. 10.3758/bf03193146 17695343

[B14] Gangarossa G, Valjent E (2012) Regulation of the ERK pathway in the dentate gyrus by in vivo dopamine D1 receptor stimulation requires glutamatergic transmission. Neuropharmacology 63:1107–1117. 10.1016/j.neuropharm.2012.06.062 22796106

[B15] Gaytan O, Swann A, Dafny N (1998) Time-dependent differences in the rat’s motor response to amphetamine. Pharmacol Biochem Behav 59:459–467. 10.1016/S0091-3057(97)00438-3 9476996

[B16] Goldberg JA, Wilson CJ (2010) The cholinergic interneurons of the striatum: intrinsic properties underlie multiple discharge patterns. Hbk Behav Neurosci 20:133–149.

[B17] Gonzales KK, Smith Y (2015) Cholinergic interneurons in the dorsal and ventral striatum: anatomical and functional considerations in normal and diseased conditions. Ann NY Acad Sci 1349:1–45. 10.1111/nyas.12762 25876458 PMC4564338

[B18] Goodwin JS, Larson GA, Swant J, Sen N, Javitch JA, Zahniser NR, De Felice LJ, Khoshbouei H (2009) Amphetamine and methamphetamine differentially affect dopamine transporters in vitro and in vivo. J Biol Chem 284:2978–2989. 10.1074/jbc.M805298200 19047053 PMC2631950

[B19] Granado N, Ares-Santos S, Tizabi Y, Moratalla R (2018) Striatal reinnervation process after acute methamphetamine-induced dopaminergic degeneration in mice. Neurotox Res 34:627–639. 10.1007/s12640-018-9925-z 29934756

[B20] Hnasko TS, Chuhma N, Zhang H, Goh GY, Sulzer D, Palmiter RD, Rayport S, Edwards RH (2010) Vesicular glutamate transport promotes dopamine storage and glutamate corelease in vivo. Neuron 65:643–656. 10.1016/j.neuron.2010.02.012 20223200 PMC2846457

[B21] Holt DJ, Hersh LB, Saper CB (1996) Cholinergic innervation in the human striatum: a three-compartment model. Neuroscience 74:67–87. 10.1016/0306-4522(96)00094-2 8843078

[B22] Jayanthi S, Torres OV, Ladenheim B, Cadet JL (2020) A single prior injection of methamphetamine enhances methamphetamine self-administration (SA) and blocks SA-induced changes in DNA methylation and mRNA expression of potassium channels in the rat nucleus accumbens. Mol Neurobiol 57:1459–1472. 10.1007/s12035-019-01830-3 31758400 PMC7060962

[B23] Jing L, Liu B, Zhang M, Liang JH (2018) Involvement of dopamine D2 receptor in a single methamphetamine-induced behavioral sensitization in C57BL/6J mice. Neurosci Lett 681:87–92. 10.1016/j.neulet.2018.02.067 29501686

[B24] Jones S, Kauer JA (1999) Amphetamine depresses excitatory synaptic transmission via serotonin receptors in the ventral tegmental area. J Neurosci 19:9780–9787. 10.1523/JNEUROSCI.19-22-09780.1999 10559387 PMC6782955

[B25] Kalivas PW, Stewart J (1991) Dopamine transmission in the initiation and expression of drug- and stress-induced sensitization of motor activity. Brain Res Brain Res Rev 16:223–244. 10.1016/0165-0173(91)90007-u 1665095

[B26] Kameda SR, Fukushiro DF, Trombin TF, Procópio-Souza R, Patti CL, Hollais AW, Calzavara MB, Abílio VC, Ribeiro RA, Tufik S, D’Almeida V, Frussa-Filho R (2011) Adolescent mice are more vulnerable than adults to single injection-induced behavioral sensitization to amphetamine. Pharmacol Biochem Behav 98:320–324. 10.1016/j.pbb.2011.01.013 21277887

[B27] Kelley AE (2001) Measurement of rodent stereotyped behavior. Curr Protoc Neurosci Chapter 8:Unit 8.8. 10.1002/0471142301.ns0808s04 18428549

[B28] Kharkwal G, Brami-Cherrier K, Lizardi-Ortiz JE, Nelson AB, Ramos M, Del Barrio D, Sulzer D, Kreitzer AC, Borrelli E (2016) Parkinsonism driven by antipsychotics originates from dopaminergic control of striatal cholinergic interneurons. Neuron 91:67–78. 10.1016/j.neuron.2016.06.014 27387649 PMC4939839

[B29] Knight ZA, Tan K, Birsoy K, Schmidt S, Garrison JL, Wysocki RW, Emiliano A, Ekstrand MI, Friedman JM (2012) Molecular profiling of activated neurons by phosphorylated ribosome capture. Cell 151:1126–1137. 10.1016/j.cell.2012.10.039 23178128 PMC3839252

[B30] Koob GF, Le Moal M (2001) Drug addiction, dysregulation of reward, and allostasis. Neuropsychopharmacology 24:97–129. 10.1016/S0893-133X(00)00195-0 11120394

[B31] Krasnova IN, Ladenheim B, Jayanthi S, Oyler J, Moran TH, Huestis MA, Cadet JL (2001) Amphetamine-induced toxicity in dopamine terminals in CD-1 and C57BL/6J mice: complex roles for oxygen-based species and temperature regulation. Neuroscience 107:265–274. 10.1016/s0306-4522(01)00351-7 11731100

[B32] Krasnova IN, Ladenheim B, Cadet JL (2005) Amphetamine induces apoptosis of medium spiny striatal projection neurons via the mitochondria-dependent pathway. FASEB J 19:851–853. 10.1096/fj.04-2881fje 15731293

[B33] Lee JH, Ribeiro EA, Kim J, Ko B, Kronman H, Jeong YH, Kim JK, Janak PH, Nestler EJ, Koo JW, Kim JH (2020) Dopaminergic regulation of nucleus accumbens cholinergic interneurons demarcates susceptibility to cocaine addiction. Biol Psychiatry 88:746–757. 10.1016/j.biopsych.2020.05.003 32622465 PMC7584775

[B34] Lewis RG, Borrelli E (2020) A mechanism of cocaine addiction susceptibility through D2 receptor-mediated regulation of nucleus accumbens cholinergic interneurons. Biol Psychiatry 88:738–740. 10.1016/j.biopsych.2020.08.022 33092690 PMC7971775

[B35] Li MH, Underhill SM, Reed C, Phillips TJ, Amara SG, Ingram SL (2017) Amphetamine and methamphetamine increase NMDAR-GluN2B synaptic currents in midbrain dopamine neurons. Neuropsychopharmacology 42:1539–1547. 10.1038/npp.2016.278 27976681 PMC5436114

[B36] Lim SA, Kang UJ, McGehee DS (2014) Striatal cholinergic interneuron regulation and circuit effects. Front Synaptic Neurosci 6:22. 10.3389/fnsyn.2014.00022 25374536 PMC4204445

[B37] Matamales M, Götz J, Bertran-Gonzalez J (2016a) Quantitative imaging of cholinergic interneurons reveals a distinctive spatial organization and a functional gradient across the mouse striatum. PLoS One 11:e0157682. 10.1371/journal.pone.0157682 27314496 PMC4912095

[B38] Matamales M, Skrbis Z, Hatch RJ, Balleine BW, Götz J, Bertran-Gonzalez J (2016b) Aging-related dysfunction of striatal cholinergic interneurons produces conflict in action selection. Neuron 90:362–373. 10.1016/j.neuron.2016.03.006 27100198

[B39] Miller DB, O’Callaghan JP (1996) Neurotoxicity of d-amphetamine in the C57BL/6J and CD-1 mouse. Interactions with stress and the adrenal system. Ann NY Acad Sci 801:148–167. 10.1111/j.1749-6632.1996.tb17438.x 8959030

[B40] Mingote S, Chuhma N, Kusnoor SV, Field B, Deutch AY, Rayport S (2015) Functional connectome analysis of dopamine neuron glutamatergic connections in forebrain regions. J Neurosci 35:16259–16271. 10.1523/JNEUROSCI.1674-15.2015 26658874 PMC4682788

[B41] Mingote S, Chuhma N, Kalmbach A, Thomsen GM, Wang Y, Mihali A, Sferrazza C, Zucker-Scharff I, Siena AC, Welch MG, Lizardi-Ortiz J, Sulzer D, Moore H, Gaisler-Salomon I, Rayport S (2017) Dopamine neuron dependent behaviors mediated by glutamate cotransmission. Elife 6:e27566. 10.7554/eLife.27566 28703706 PMC5599237

[B42] Mingote S, Amsellem A, Kempf A, Rayport S, Chuhma N (2019) Dopamine-glutamate neuron projections to the nucleus accumbens medial shell and behavioral switching. Neurochem Int 129:104482. 10.1016/j.neuint.2019.104482 31170424 PMC6855309

[B101] Paxinos G, Franklin KBJ (2008) The mouse brain in stereotaxic coordinates, Ed 3. San Diego:Elsevier/Academic Press.

[B43] Phelps PE, Vaughn JE (1986) Immunocytochemical localization of choline acetyltransferase in rat ventral striatum: a light and electron microscopic study. J Neurocytol 15:595–617. 10.1007/BF01611860 3534148

[B44] Rapanelli M, Frick LR, Pogorelov V, Ota KT, Abbasi E, Ohtsu H, Pittenger C (2014) Dysregulated intracellular signaling in the striatum in a pathophysiologically grounded model of Tourette syndrome. Eur Neuropsychopharmacol 24:1896–1906. 10.1016/j.euroneuro.2014.10.007 25464894 PMC4306602

[B45] Ricaurte GA, Seiden LS, Schuster CR (1984) Further evidence that amphetamines produce long-lasting dopamine neurochemical deficits by destroying dopamine nerve fibers. Brain Res 303:359–364. 10.1016/0006-8993(84)91221-6 6744029

[B46] Rincón-Cortés M, Gagnon KG, Dollish HK, Grace AA (2018) Diazepam reverses increased anxiety-like behavior, social behavior deficit, and dopamine dysregulation following withdrawal from acute amphetamine. Neuropsychopharmacology 43:2418–2425. 10.1038/s41386-018-0123-8 29959439 PMC6180061

[B47] Robinson TE (1984) Behavioral sensitization: characterization of enduring changes in rotational behavior produced by intermittent injections of amphetamine in male and female rats. Psychopharmacology (Berl) 84:466–475. 10.1007/BF00431451 6441946

[B48] Robinson TE, Becker JB (1986) Enduring changes in brain and behavior produced by chronic amphetamine administration: a review and evaluation of animal models of amphetamine psychosis. Brain Res 396:157–198. 10.1016/s0006-8993(86)80193-7 3527341

[B49] Russo SJ, Dietz DM, Dumitriu D, Morrison JH, Malenka RC, Nestler EJ (2010) The addicted synapse: mechanisms of synaptic and structural plasticity in nucleus accumbens. Trends Neurosci 33:267–276. 10.1016/j.tins.2010.02.002 20207024 PMC2891948

[B50] Ryan LJ, Linder JC, Martone ME, Groves PM (1990) Histological and ultrastructural evidence that D-amphetamine causes degeneration in neostriatum and frontal cortex of rats. Brain Res 518:67–77. 10.1016/0006-8993(90)90955-b 1975218

[B51] Sofuoglu M, Mooney M (2009) Cholinergic functioning in stimulant addiction: implications for medications development. CNS Drugs 23:939–952. 10.2165/11310920-000000000-00000 19845415 PMC2778856

[B52] Stuber GD, Hnasko TS, Britt JP, Edwards RH, Bonci A (2010) Dopaminergic terminals in the nucleus accumbens but not the dorsal striatum corelease glutamate. J Neurosci 30:8229–8233. 10.1523/JNEUROSCI.1754-10.2010 20554874 PMC2918390

[B53] Sulzer D (2011) How addictive drugs disrupt presynaptic dopamine neurotransmission. Neuron 69:628–649. 10.1016/j.neuron.2011.02.010 21338876 PMC3065181

[B54] Underhill SM, Hullihen PD, Chen J, Fenollar-Ferrer C, Rizzo MA, Ingram SL, Amara SG (2021) Amphetamines signal through intracellular TAAR1 receptors coupled to Gα_13_ and Gα_S_ in discrete subcellular domains. Mol Psychiatry 26:1208–1223. 10.1038/s41380-019-0469-2 31399635 PMC7038576

[B55] Valjent E, Bertran-Gonzalez J, Bowling H, Lopez S, Santini E, Matamales M, Bonito-Oliva A, Hervé D, Hoeffer C, Klann E, Girault JA, Fisone G (2011) Haloperidol regulates the state of phosphorylation of ribosomal protein S6 via activation of PKA and phosphorylation of DARPP-32. Neuropsychopharmacology 36:2561–2570. 10.1038/npp.2011.144 21814187 PMC3194082

[B56] Vanderschuren LJ, Schmidt ED, De Vries TJ, Van Moorsel CA, Tilders FJ, Schoffelmeer AN (1999) A single exposure to amphetamine is sufficient to induce long-term behavioral, neuroendocrine, and neurochemical sensitization in rats. J Neurosci 19:9579–9586. 10.1523/JNEUROSCI.19-21-09579.1999 10531460 PMC6782918

[B57] Wagner GC, Ricaurte GA, Johanson CE, Schuster CR, Seiden LS (1980) Amphetamine induces depletion of dopamine and loss of dopamine uptake sites in caudate. Neurology 30:547–550. 10.1212/wnl.30.5.547 6768005

[B58] Witten IB, Lin SC, Brodsky M, Prakash R, Diester I, Anikeeva P, Gradinaru V, Ramakrishnan C, Deisseroth K (2010) Cholinergic interneurons control local circuit activity and cocaine conditioning. Science 330:1677–1681. 10.1126/science.1193771 21164015 PMC3142356

[B59] Wolf ME, Xue CJ (1998) Amphetamine and D1 dopamine receptor agonists produce biphasic effects on glutamate efflux in rat ventral tegmental area: modification by repeated amphetamine administration. J Neurochem 70:198–209. 10.1046/j.1471-4159.1998.70010198.x 9422363

[B60] Xia YF, He L, Whistler JL, Hjelmstad GO (2008) Acute amphetamine exposure selectively desensitizes kappa-opioid receptors in the nucleus accumbens. Neuropsychopharmacology 33:892–900. 10.1038/sj.npp.1301463 17551543 PMC2268619

[B61] Xue CJ, Ng JP, Li Y, Wolf ME (1996) Acute and repeated systemic amphetamine administration: effects on extracellular glutamate, aspartate, and serine levels in rat ventral tegmental area and nucleus accumbens. J Neurochem 67:352–363. 10.1046/j.1471-4159.1996.67010352.x 8667013

[B62] Yates JW, Meij JT, Sullivan JR, Richtand NM, Yu L (2007) Bimodal effect of amphetamine on motor behaviors in C57BL/6 mice. Neurosci Lett 427:66–70. 10.1016/j.neulet.2007.09.011 17920769 PMC2117340

[B63] Zhu JP, Xu W, Angulo JA (2006) Methamphetamine-induced cell death: selective vulnerability in neuronal subpopulations of the striatum in mice. Neuroscience 140:607–622. 10.1016/j.neuroscience.2006.02.055 16650608 PMC2882192

